# Systematic review of *Anopheles* abundance and meta-analysis of nonhuman primate malaria infection in mosquitoes in Thailand

**DOI:** 10.1186/s13071-026-07414-0

**Published:** 2026-05-16

**Authors:** Sampath N. Weerakoon, Jeffrey Hii, Jirod Nararak, Chutipong Sukkanon, Ratchadawan Ngoen-Klan, Uraiwan Arunyawat, Pattarapol Maneeon, Sorawat Thongsahuan, Indra Vythilingam, Sylvie Manguin, Theeraphap Chareonviriyaphap

**Affiliations:** 1 Department of Entomology, Faculty of Agriculture, Kasetsart University, Bangkok, 10900 Thailand; 2 College of Public Health, Medical and Veterinary Sciences, James Cook University, Townsville, QLD Australia; 3 Department of Clinical Microscopy, Faculty of Medical Technology, Mahidol University, Nakhon Pathom, 73170 Thailand; 4 Department of Genetics, Faculty of Science, Kasetsart University, Bangkok, 10900 Thailand; 5 Ministry of Natural Resources and Environment, Department of National Parks, Wildlife and Plant Conservation, Bangkok, 10900 Thailand; 6 Faculty of Veterinary Science, Prince of Songkla University, Songkhla, 90110 Thailand; 7 Department of Parasitology, Faculty of Medicine, Universiti Malaya, Kuala Lumpur, Malaysia; 8 HSM, Univ. Montpellier, CNRS, Montpellier, IRD France; 9 Research and Lifelong Learning Centre for Urban and Environmental Entomology, Kasetsart University Institute for Advanced Studies, Kasetsart University, Bangkok, 10900 Thailand

**Keywords:** *Anopheles*, Mosquito infectivity, Thailand, Nonhuman primate malaria

## Abstract

**Background:**

Thailand has achieved significant progress in malaria elimination, with a reduction in annual parasite incidence from 0.53 to 0.22 per thousand in 2014 and 2024, respectively. Given the high diversity of *Anopheles* mosquito species, elimination efforts must be precisely targeted, taking into account the varied behaviors and vectorial capacities of different vector species. This study aims to systematically review and update the distribution, identification, bionomics, behavior, and a meta-analysis of nonhuman parasite infectivity among mosquitoes.

**Methods:**

A comprehensive literature search was conducted in PubMed, Scopus, EBSCOhost, and Google Scholar (2013–2025) to identify studies on *Anopheles* species diversity, distribution, and zoonotic malaria infection in mosquitoes. The meta-analysis followed Preferred Reporting Items for Systematic Reviews and Meta-Analyses (PRISMA) guidelines and was carried out using the metafor package in R.

**Results:**

A total of 92 relevant papers were included from 811 accessed articles. Of these, most documented geographical distribution, followed by mosquito behaviors, molecular identification, and mosquito infectivity. The pooled mosquito infection prevalence for the present meta-analysis was 0.01 (95% confidence interval (CI) 0.00–0.03), indicating low nonhuman primate (NHP) malaria parasite infectivity. Using standard nested polymerase chain reaction (PCR), wild-caught *Anopheles sawadwongporni*, *An. minimus*, and *An. dirus* were incriminated as zoonotic malaria vectors, with a pooled infection prevalence of 0.2%.

**Conclusions:**

This review highlights a critical need for targeted, context-specific vector control interventions that address the unique opportunistic feeding and resting behaviors of *Anopheles* species complexes and the bionomics of local vector species, alongside the high mobility of high-risk populations. Research involving hosts and vectors across different micro-spatial and temporal scales, observations of human behavior, and monkey–human interactions is needed to improve understanding of zoonotic malaria transmission.

**Graphical Abstract:**

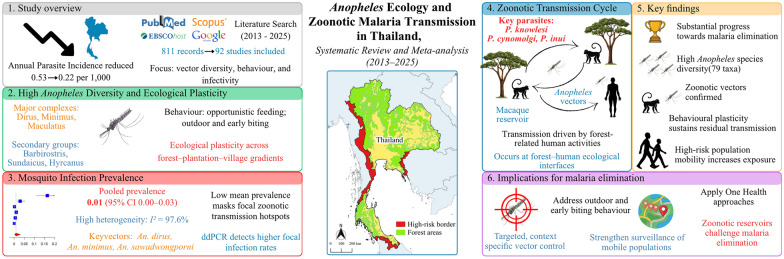

## Background

Mosquitoes of the genus *Anopheles* are the only known vectors capable of transmitting malaria-causing *Plasmodium* parasites to humans. In Thailand, 79 *Anopheles* taxa have been reported, comprising 77 formally described species and two unnamed presumptive species [1]. Although many taxa occur within morphologically similar species complexes, only a limited number are recognized as primary or secondary malaria vectors [1]. These include species within the Dirus complex (i.e., *An. dirus* Peyton & Harrison and *An. baimaii* Sallum & Peyton), the Funestus group (i.e., *An. minimus* Theobald and *An. aconitus* Doenitz), and the Maculatus group (i.e., *An. maculatus* Theobald, *An. sawadwongporni* Rattanarithikul & Green, and *An. pseudowillmori* (Theobald)). Additional taxa within the Barbirostris and Sundaicus complexes and the Hyrcanus group are regarded as secondary or suspected vectors, reflecting uncertainty in their epidemiological roles and highlighting the importance of accurate species identification and evidence synthesis [1–3].

Thailand is one of eight countries identified by the World Health Organization (WHO) as having complex malaria transmission while remaining on a trajectory toward elimination by 2025 [4]. Although indigenous cases declined substantially from 32,480 in 2010 to 22,426 in 2021, recent increases from 6263 in 2022 to 9169 in 2023 have been observed, particularly along internationbal borders [5]. This resurgence has been driven by renewed *Plasmodium falciparum* transmission along the Thai–Myanmar border and a rising incidence of zoonotic *Plasmodium knowlesi* along the Thai–Malaysian and Thai–Cambodian borders [5]. Confirmed *P. knowlesi* cases increased from a single case in 2016 to 259 cases in 2023, with an additional 78 cases reported in 2024 [6].

The emergence and spread of NHP malaria, particularly *P. knowlesi* and *P. cynomolgi*, in Southeast Asian countries are strongly influenced by landscape change. Human-driven land-use alterations, including deforestation and conversion of forested areas to agricultural plantations, are major drivers of NHP malaria transmission, especially of these two zoonotic malaria species [7–9]. Such environmental changes create favorable conditions for *Anopheles* vectors by increasing vector abundance and facilitating closer spatial overlap between humans, macaque hosts, and mosquito populations [10]. Modified environments, particularly plantations, generate microclimatic conditions characterized by increased humidity, reduced wind, and shaded breeding sites that enhance vector survival and productivity [10–12]. Together, these dynamics highlight the importance of integrated One Health approach that address ecological, entomological, human, and simian behavioral drivers of NHP malaria in rapidly changing landscapes [7].

Human-driven landscape modifications, particularly deforestation, create ecological interfaces in which *Anopheles* mosquitoes and macaque monkeys, the natural reservoir hosts of *P. knowlesi*, coexist with human populations [13]. These altered environments promote zoonotic spillover by increasing spatial and temporal overlap among vectors, nonhuman primate hosts, and humans. In densely populated areas or intensively used areas adjacent to macaque habitats, this overlap increases mosquito–macaque contact rates and facilitates the transmission of malaria parasites from macaques to humans [10, 14].

Previous research on malaria in Thailand has primarily described epidemiological patterns, including prevalence or incidence, morbidity, and mortality associated with human *Plasmodium* parasites and the zoonotic parasite *P. knowlesi* [15]. Parallel advances in *Anopheles* systematics have underscored the importance of accurate vector identification for interpreting transmission dynamics and informing control strategies [1, 16]. Consequently, *Anopheles* taxonomy in Thailand has evolved from reliance on morphological characters alone to the incorporation of molecular and genetic tools. Techniques such as PCR-based assays (including AS-PCR, SCAR-PCR, RFLP-PCR, and multiplex PCR), DNA sequencing of nuclear and mitochondrial markers (ITS2, D3, and COII), and microsatellite markers have become essential for distinguishing morphologically similar sibling species that differ in vectorial capacity [1, 16, 17].

Thailand has a long history of malaria vector research, initially based exclusively on morphological identification [18–20]. A comprehensive survey conducted in 1975 documented 32 species and one subspecies [21]. Subsequent cytogenetic studies revealed the presence of isomorphic or cryptic species within several major *Anopheles* complexes and groups, including the *An. dirus* complex, the *An. maculatus* complex [22–25], the *An. hyrcanus* group [26], and the *An. barbirostris* and *An. umbrosus* species groups [27]. Building on this work, Rattanarithikul et al. [20] expanded the recognized *Anopheles* fauna in Thailand to 73 species, including newly described members within the Leucosphyrus group, the Sundaicus complex, and the Minimus complex based on morphological characters [20]. Molecular diagnostic tools, particularly multiplex PCR-based assays, were subsequently developed to facilitate routine identification of sibling species within these complexes [28–32].

Despite these advances, evidence relevant to NHP malaria transmission remains fragmented and the relative roles of different *Anopheles* species are incompletely synthesized. In particular, there has been a lack of a comprehensive, systematic integration of molecularly confirmed vector records, entomological infection data, and species-specific ecological information relevant to zoonotic malaria in Thailand. This systematic review therefore collates and critically appraises published evidence on the distribution and diversity of *Anopheles* species in Thailand, with emphasis on sibling species confirmed using molecular methods since 2013. In addition, a meta-analysis is conducted to estimate mosquito infectivity with NHP malaria parasites where data permit. By integrating these findings with recent evidence on vector bionomics, surveillance, and control, this study aims to clarify current knowledge, quantify available evidence, and identify priority gaps to support malaria elimination and management of residual and zoonotic transmission in Thailand.

## Methods

### Strategy of the literature survey

A systematic literature review was conducted following Preferred Reporting Items for Systematic Reviews and Meta-Analyses (PRISMA) guidelines [33, 34].

### Databases used and search strategy

**Fig. 1:**
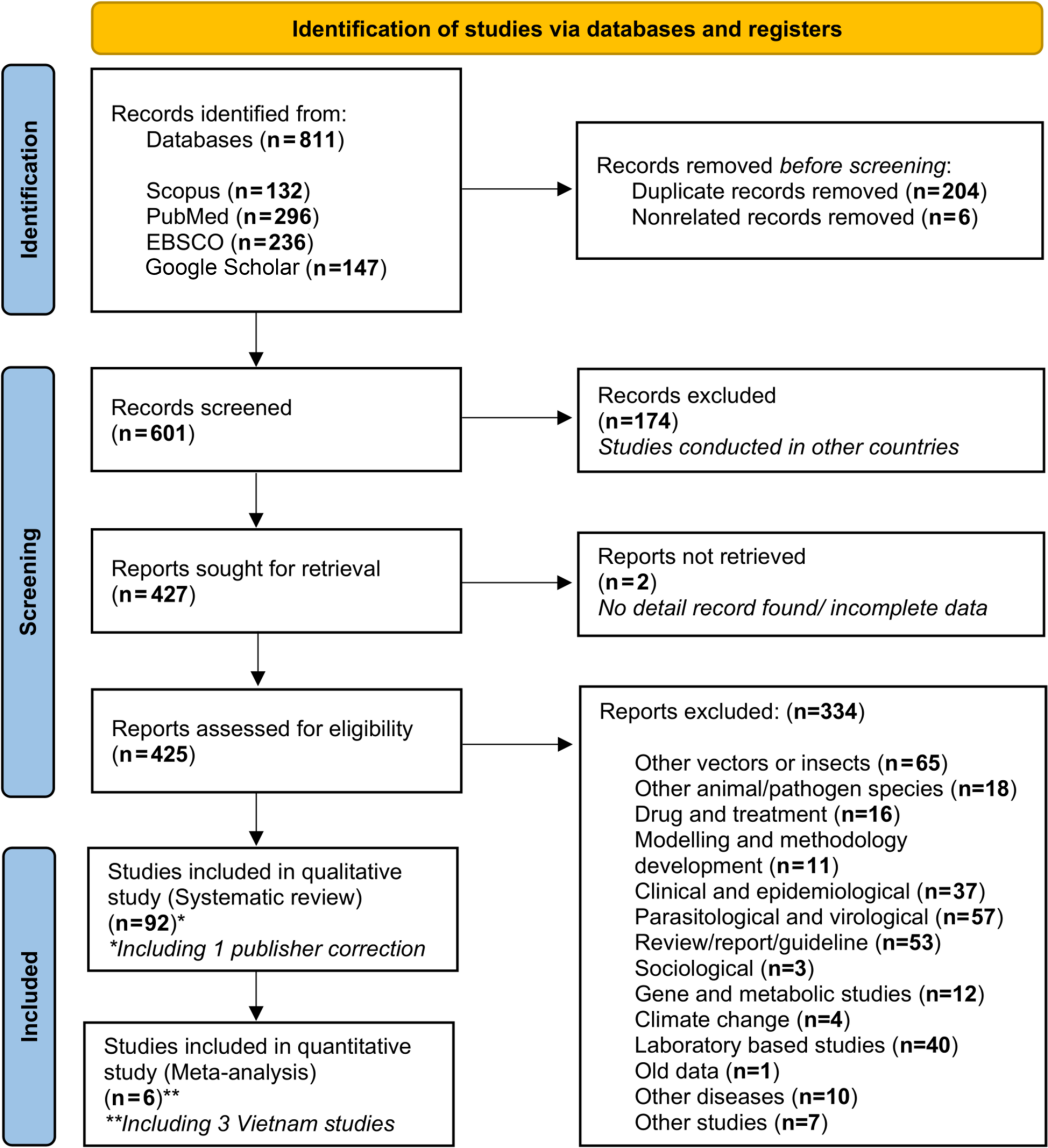
PRISMA flow diagram showing database search results with numbers of studies included/excluded at each stage

Peer-reviewed, published literature was reviewed using four electronic bibliographic databases: PubMed, Scopus, EBSCOhost, and Google Scholar. In addition, manual Google searches and screening of reference lists were performed to identify additional articles (Fig. 1).

Articles published from 2013 to 2025 were searched using the term “*Anopheles*” in combination with the Boolean operators AND/OR and the following keywords: “diversity,” “composition,” “*Plasmodium*,” “vector infectivity,” “malaria,” “simian malaria,” “knowlesi malaria,” and “Thailand” or “Thai” or “Greater Mekong Subregion.” Studies that only cited another article’s collections were excluded. All articles were exported as .csv files for Microsoft Excel, and duplicates were removed manually. EndNote version 21.5 was used to manage and cite references.

All studies were screened for relevance to the meta-analysis of mosquito sporozoite infections and scored as follows: (1) studies meeting all inclusion criteria (see below), (2) studies meeting all inclusion criteria except for some components required for the meta-analysis, or (3) irrelevant studies. The final numbers of studies included in the meta-analysis and the systematic review were 6 and 92, respectively (Fig. 1).

### Scientific literature screening and data extraction

All selected articles were in English. Documents not fulfilling the following criteria were excluded: original research addressing bionomics, malaria parasite infectivity, insecticide resistance, or prevention and control of *Anopheles* vectors, with full-text availability. Reviewed documents included review and research articles, conference abstracts, mini-reviews, and short communications.

Data extraction was performed on articles that met these inclusion criteria: malaria parasite detection in *Anopheles* natural populations and infection or transmission in laboratory-reared mosquitoes. The following variables were compiled from each article: main author, publication date, study type (field, semi-field, or laboratory), study location, collection date, *Anopheles* species studied, mosquito sex, number of mosquitoes analyzed, human biting or landing rates, resting sites, number of mosquitoes per pool, number of pools positive for malaria parasites, parasite detection method, *Plasmodium* species, and taxonomic assignment.

### Statistical analysis

All analyses were conducted in R version 4.5.1. A random-effects model meta-analysis was used to estimate the prevalence of *P. knowlesi* in *Anopheles* mosquitoes. The “metaprop” function in the “meta” package was used to calculate logit-transformed pooled prevalence and 95.0% confidence intervals, and results were visualized using the “forestplot” package in RStudio [35]. Heterogeneity was assessed using the *I*^2^ statistic; higher values indicate greater heterogeneity across studies. Because individual study weights were not calculated, publication bias was not assessed [36].

## Results

### Molecular biosystematics and population genetic structure of *Anopheles* sibling complexes

Multiplex PCR assays targeting ITS2 are widely used to identify cryptic species within complexes or groups, including the Dirus complex (*An. dirus*, *An. baimaii*, *An. cracens*, *An. nemophilous*, and *An. scanloni*) [32, 37], the Maculatus group (*An. maculatus* s.s., *An. dravidicus*, *An. pseudowillmori*, *An. rampae*, and *An. sawadwongporni*) [37, 38], the Funestus group (*An. minimus*, *An. harrisoni*, *An. aconitus*, *An. pampanai*, and *An. varuna*) [29, 37, 39], and the Barbirostris complex (*An. barbirostris*, *An. dissidens*, *An. saeungae*, *An. wejchoochotei*, and *An. barbirostris* A3) [30, 40, 41]. A new primer set for Dirus complex species identification PCR (DiCSIP) has been developed to improve the specificity, operational range, and sensitivity for five complex members in the Greater Mekong Subregion. The assay was validated using field-collected specimens morphologically identified as the *An. dirus* complex, with ten specimens from each of four Thai provinces: Kanchanaburi, Prachinburi, Ranong, and Sisaket [32]. DiCSIP provides advantages over older AS-PCR assays, including improved specificity, efficiency, and sensitivity, yielding more reliable and reproducible results. Older AS-PCR assays may not represent the full genetic diversity or evolutionary changes in ITS2 sequences, whereas DiCSIP uses primers designed from up-to-date sequence information [32].

DNA sequencing studies are crucial for understanding mosquito genetic diversity, population structure, and gene function through assessments of variation within and between populations. Nucleotide diversity varies among *An. epiroticus*, *An. minimus*, and *An. maculatus* populations [37]. Another study suggested high genetic distances among species within a clade of the *Hyrcanus* group [42]. Sequencing of specific genes such as voltage-gated sodium channel enables detection of mutations associated with insecticide resistance; however, no evidence of *kdr* mutations was found in sampled *Anopheles* populations in *P. knowlesi*-endemic areas in Thailand, suggesting metabolic resistance [37, 43].

Studies using mitochondrial DNA markers such as COI and COII revealed that current populations of *An. minimus* in Thailand are divided into two lineages, A and B [37]. Lineage A is characterized by high haplotype diversity and signals of population expansion, suggesting a high degree of adaptability [44]. A recent analysis of the mitochondrial COI gene in island populations of *An. baimaii* in Thailand showed low nucleotide diversity but high haplotype diversity [45]. Analysis of the COI gene of *An. epiroticus* revealed two distinct genetic clades in Ranong Province, Thailand, indicating variation potentially linked to local adaptation [37]. Mitochondrial DNA markers are effective for species identification owing to conserved mitochondrial genes with high copy numbers and a distinct “barcoding gap,” which enables clear species differentiation and supports the identification of unknown, ambiguous, or damaged specimens when morphology is unreliable [41].

### Primary vectors: *dirus* and *minimus* complexes and Maculatus group

#### *Anopheles* (Cellia) *dirus* Peyton & Harrison species complex (Dirus complex)

**Fig. 2:**
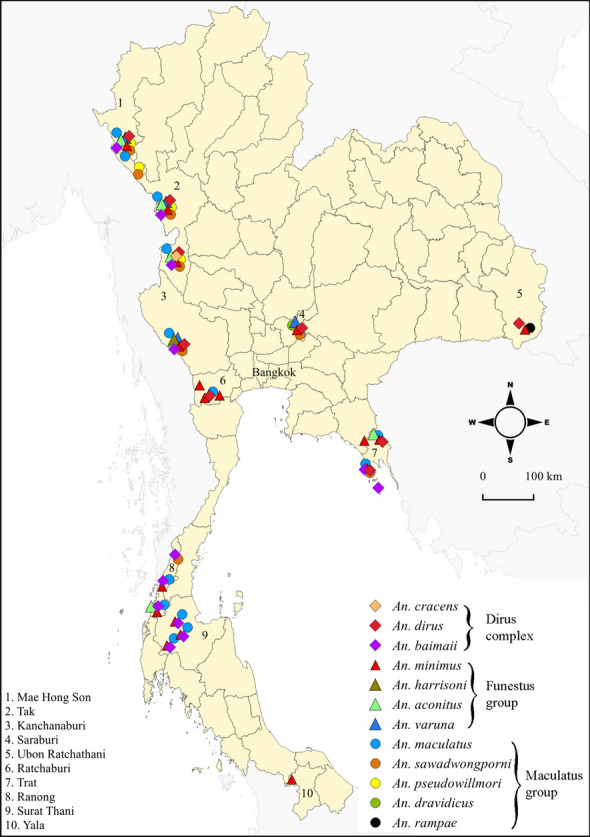
Distribution of molecularly confirmed species of the *Anopheles dirus* complex (diamonds), Funestus group (triangles), and Maculatus group (circles) based on studies from 2013–2025

Mosquito species diversity was highest along Thailand’s international border regions with Myanmar, Laos, and Cambodia. This review highlights provinces including Saraburi, Trat, Ubon Ratchathani, Mae Hong Son, Tak, Ratchaburi, and Kanchanaburi as having particularly diverse *Anopheles* fauna (Fig. 2).

During the wet season, *An. dirus* populations showed peak activity in Ubon Ratchathani Province in July 2014 and September 2015 [46]. Similarly, in Umphang Valley, Tak Province, the total number of *An. dirus* collected (*n* = 24) was high in the wet season (from August to October), with densities dropping to as low as two specimens by December [47]. The seasonal patterns of *An. dirus* s.l. populations in Thailand remain consistent with the previous review [15], with highest abundance during the wet season. In these areas, adult densities of *An. dirus* s.l. were positively associated with increased rainfall from July to August/September, as previously observed [15, 48].

Changes in land use due to anthropogenic activities, such as expansion of human settlements, forest fragmentation through logging, and deforestation for rice cultivation, cassava, sugar, and rubber, have created conditions favorable for the proliferation of primary malaria vectors in Thailand [10, 49]. Human activities within these altered environments increase *An. dirus* breeding opportunities; for example, puddles on muddy roads act as temporary freshwater larval habitats after rainfall [10]. These changes contribute to increased malaria transmission risk, especially in southern Thailand [50]. *An. dirus* demonstrates adaptability to land-use changes, such as the conversion of rice paddies to mature rubber plantations [46]. This adaptability allows the species to persist and proliferate in altered environments, particularly in hilly areas adjacent to natural forests [11, 51]. In Sisaket Province, *An. dirus* occurred in both rubber–forest and village ecotypes, with higher biting density in forest sites. Rubber tappers in plantations near forests, as well as forest goers, are therefore at greater risk [52]. Biting activity was also slightly higher during the rainy and cool-dry seasons compared with the hot-dry season [52]. The larval habitats of this species are typically small, shallow, shaded bodies of fresh, stagnant, or slow-flowing water, remaining consistent since early reviews [53]. During the dry season, these habitats contracted to subterranean seepage streams and wells, as first noted by Rosenberg et al. (1990) [54] and more recently by Saeung et al. (2025) [52]. This shift suggests that females face difficulty locating suitable breeding sites during the dry season owing to their scarcity or unsuitability for larval development [54].

Distributions of molecularly identified *An. baimaii* were recorded at varying densities in six provinces: Trat, Mae Hong Son, Tak, Kanchanaburi, Surat Thani, and Ranong (Fig. 2). Additionally, *An. baimaii* was first reported from four islands, including Chang and Kood islands in Trat Province, along the Thai–Cambodian border, and Chang and Phayam islands in Ranong Province, adjacent to the Thai–Myanmar border. This extends the known range of *An. baimaii* to the easternmost islands [55]. While earlier studies reported *An. baimaii* in western Thailand, current data confirm its presence along both the western and eastern borders [15, 56].

The presence of *An. baimaii* on these border islands exemplifies “anophelism without malaria” [57], underscoring the need for continuous monitoring. Geographical isolation of island populations appears to influence morphological and genetic variation, with a mean genetic variation of 0.73 between populations on Kood Island (Trat Province) and Chang Island (Ranong Province) [45]. The southern region of Thailand and the Thai–Myanmar border are locations of high sympatry for several Dirus complex species [56], particularly *An. baimaii* in the western regions [17].

In a survey in Surat Thani Province, molecular analysis of *An. dirus* s.l. specimens revealed the exclusive presence of *An. baimaii* (100.0%, *n* = 348). This was the first record of the species in this region, suggesting prior misidentification using morphology alone [51]. These and other recent molecular studies have improved understanding of *An. baimaii* distribution. The species has been confirmed in northwestern Thailand, including Mae Sot and Sop Moei [58] and Umphang Valley [59], although often at low densities. This represents a new record in Surat Thani, southern Thailand, despite the occurrence of other Dirus complex species in nearby southern provinces [51].

#### *Anopheles* (Cellia) *minimus* Theobald species complex (Minimus complex)

*An. minimus*, previously identified as *An. minimus* species A, is recognized as a major malaria vector throughout the Greater Mekong Subregion (GMS), including Thailand [44]. In contrast to *An. dirus*, *An. minimus* has a more widespread distribution, with habitats that include forest-edge areas [17, 37, 60], hilly forested regions [61], and rubber plantations, where it can be dominant [11]. These findings suggest that *An. minimus* habitats have changed over the past few decades, shifting from traditional breeding sites such as slow-moving streams, rice fields, and ground pools [53] to new habitats. Rubber plantations often contain a mosaic of temporary pools, drainage ditches, and other manmade water sources that are ideal for *An. minimus* and *An. dirus* [11]. This species is more commonly found in areas of high malaria transmission, particularly in high-risk areas along Thailand’s western and eastern international borders [44]. Its occurrence aligns with the current or recent malaria microstratification by the Bureau of Vector-Borne Diseases, Ministry of Public Health [6] in areas that overlap with locations where annual average cases exceed 56 per year and with the reported distribution of *An. minimus* from the northeastern border (Ubon Ratchathani, Chanthaburi, and Trat provinces), the northwestern region (Kanchanaburi, Tak, Mae Hong Son, and Ratchaburi), and the south-western region in Ranong Province, especially in the northern and southern parts of the province (Fig. 2).

In northwestern Thailand, *An. minimus* was the most abundant *Anopheles* species recorded in Tak and Mae Hong Son provinces during a 2-year collection period (February 2011 to January 2013), representing 43.0% of total *Anopheles* captures [58]. Similarly, in southern Thailand (Surat Thani Province), *An. minimus* s.l. was the most abundant *Anopheles* species across three districts (Phanom, Khiri Rat Nikhom, and Vibhavadi), representing 69.9–93.8% of collections per district [51]. A study in western Thailand detected native *Wolbachia pipientis* in *An. minimus* collected from a low-transmission area in Umphang Valley, Tak Province, along the Thai–Myanmar border [59]. The discovery of a naturally occurring *Wolbachia* strain in wild-caught *Anopheles* necessitates further research to identify the specific strain, determine its prevalence in the wild population, and assess its potential impact on malaria transmission [59, 62].

*An. harrisoni* is a cryptic sibling species within the *An. minimus* complex and a member of the *An. minimus* subgroup [17, 63]. It is geographically distributed in Southeast Asia [17, 64] and has been reported in Kanchanaburi Province, Thailand [65]. Morphologically, *An. harrisoni* is difficult to distinguish from *An. minimus*, and the two frequently occur in sympatry in western Thailand [66]. While *An. minimus* is an opportunistic feeder, *An. harrisoni* tends to be more zoophilic, and its precise role as a malaria vector is unclear [60]. Accurate identification requires specialized molecular methods; multiplex AS-PCR targeting ITS2 has been developed for this purpose [29]. In addition, geometric morphometrics based on wing venation geometry can differentiate *An. harrisoni* from *An. minimus* with high accuracy (up to 95.0%), although size overlap exists [66].

#### *Anopheles* (Cellia) *maculatus* Theobald species group (Maculatus group)

Thailand is home to seven species of the Maculatus group, of which three are considered primary malaria vectors: *An. maculatus* Theobald, *An. sawadwongporni* Rattanarithikul & Green, and *An. pseudowillmori* (Theobald) [1]. The complex taxonomy of this group requires ongoing revision based on available molecular techniques [38, 67] for reliable identification of sibling species, notably including *An. willmori* and the species formerly known as *An. maculatus* Form K, formally described as *An. rampae* [68].

The distribution of five of the seven *An. maculatus* group species in Thailand is shown in Fig. 2. Field studies confirm that the group’s distribution is consistent with high-transmission areas. A sporozoite rate of 0.1% for *P. vivax* in *An. maculatus* (*n* = 778) collected by indoor light traps supports evidence of malaria resurgence in Tak Province, western Thailand [69]. This resurgence saw a sevenfold increase in cases in 2023 (~ 17,000) compared with 2021, and twice the number reported in 2022 [6, 70, 71]. Research in northwestern Thailand (Mae Sot District, Tak Province, and Sop Moei District, Mae Hong Son Province), along the Thai–Myanmar border, found molecularly identified *An. maculatus* (*n* = 1436) to be the second most abundant species (20.1%) among 7129 *Anopheles* collected, after *An. minimus* (42.8%) [58]. Studies in Surat Thani and Narathiwat provinces in southern Thailand also reported the presence of *An. maculatus* s.l. (1.0% prevalence) among a total of 60 *Anopheles* captured by human landing catch [51].

In Ranong Province, a highly endemic area for *P. knowlesi* [6], molecular analysis confirmed the presence of *An. maculatus* (13.2%; *n* = 15). However, none of the collected mosquitoes were positive for *Plasmodium* [37]. Members of the *An. maculatus* group, including *An. maculatus* [*n* = 164, human landing catch (HLC) = 1.72 bites per person per night], *An. sawadwongporni* (*n* = 400, HLC = 1.32 bites per person per night), and *An. pseudowillmori* (*n* = 100, HLC = 0.86 bites per person per night), were found along the Thailand–Myanmar border [72]. Another study at the Thai–Lao border in Ubon Ratchathani Province (northeastern Thailand) found very few *An. sawadwongporni* (*n* = 3) and relatively high numbers of *An. rampae*, a nonvector (*n* = 112), suggesting a complex interplay between low-density villages that contribute to relatively low transmission and neighboring forest areas where the human biting rate of *An. sawadwongporni* is low [73].

Despite a 2014 malaria outbreak with an incidence of 3.94 per thousand linked to rosewood smuggling in Ubon Ratchathani [74] and ongoing transmission in 2018 of 0.28 per thousand [6, 75], no *Plasmodium*-infected *An. maculatus* were detected [76]. This may reflect low abundance of primary vectors, temporal mismatch between the study and the transmission season, and limitations in PCR sensitivity and specificity [76]. Transmission most likely occurred in forested, hilly areas outside villages rather than within village collection sites. Extensive entomological surveys of zoonotic malaria in Narathiwat Province, southern Thailand, found *An. maculatus* to be the most prevalent species (37.1%) among captured mosquitoes (*n* = 136), with a high human biting rate of 3.40 per person per night [77].

### Secondary vectors: Barbirostris, Sundaicus, Hyrcanus, and Subpictus groups

#### *Anopheles *(*Anopheles*)* Barbirostris* van der Wulp species complex (Barbirostris complex)

Molecular identification within the *An. barbirostris* subgroup, which comprises 11 species including 8 in the Barbirostris complex, confirmed nine cryptic species: *An. barbirostris* (0.3%, *n* = 3), *An. dissidens* (17.9%, *n* = 199), *An. saeungae* (15.2%, *n* = 168), *An. wejchoochotei* (45.4%, *n* = 503), *An. campestris* (15.5%, *n* = 172), *An. barbirostris* species A3 (0.5%, *n* = 6), *An. donaldi* (2.9%, *n* = 32), *An. hodgkini* (0.2%, *n* = 2), and *An. pollicaris* (2.1%, *n* = 23) [2, 41]. The *P. vivax* infectivity rate of *An. barbirostris* s.l. was 1.7%, with two positive specimens detected from indoor and outdoor collections along the Thai–Myanmar border [78]. This finding highlights the importance of the complex as a local vector and a significant hurdle for elimination due to its contribution to residual transmission.

**Fig. 3:**
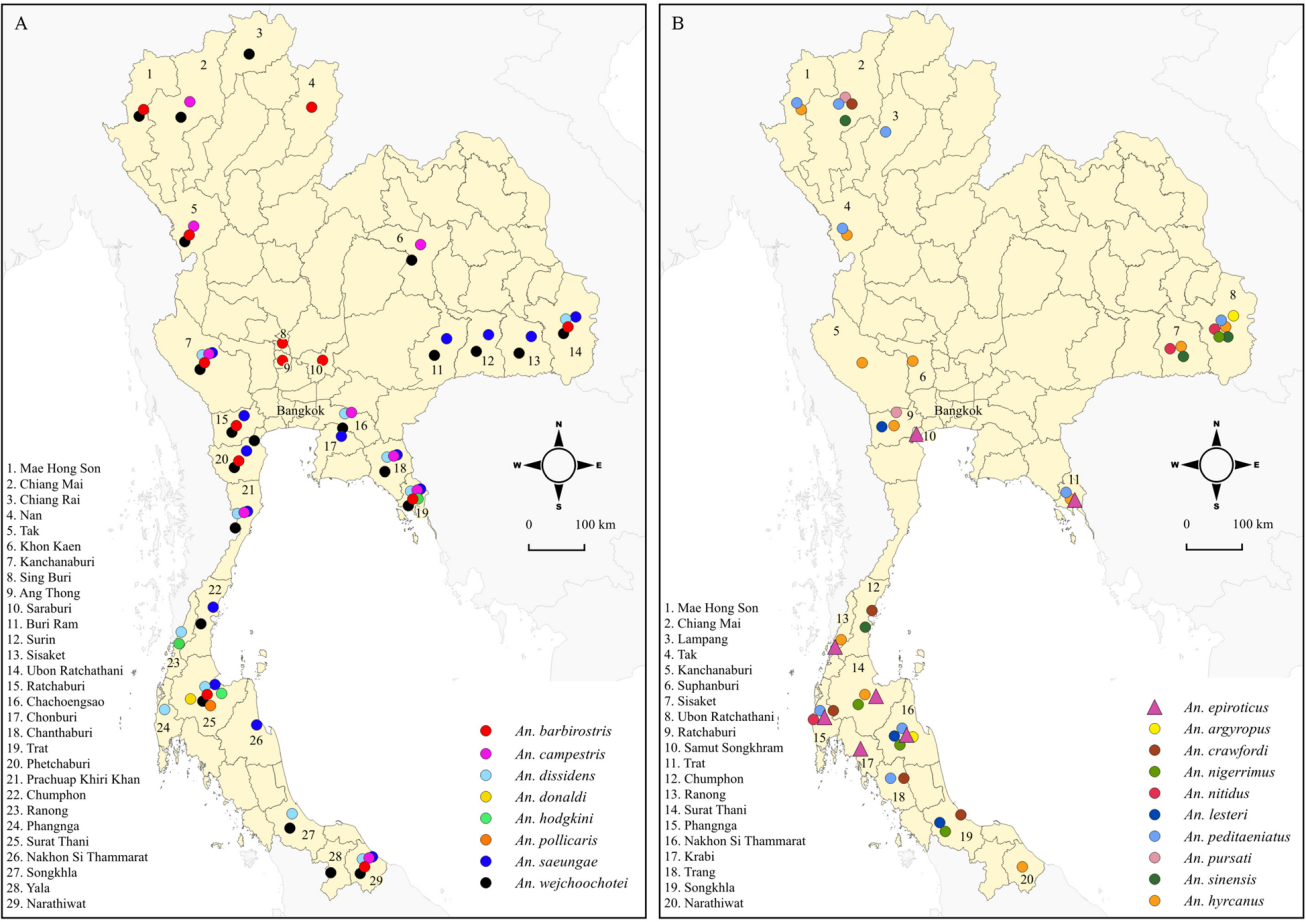
Distribution of secondary and suspected malaria vectors in Thailand based on studies from 2013–2025: **A** *Anopheles barbirostris* subgroup; **B** *Anopheles epiroticus* (triangles) and *Anopheles hyrcanus* group (circles)

The Barbirostris subgroup and complex are widely distributed in Thailand, with the exception of the north-central and northeastern regions. Population density and seasonal abundance vary geographically (Fig. 3A). Peak abundance generally occurs during the rainy and cool-dry seasons, whereas populations along the Thai–Myanmar border peak during the transition from the wet to the dry season [79]. Distribution patterns of sibling species align with high-risk malaria areas, including Mae Hong Son, Tak, Kanchanaburi, and several southern provinces [6], suggesting a strong correlation between the presence of Barbirostris species and transmission risk.

*An. wejchoochotei*, a species within the Barbirostris complex, is considered a probable malaria vector [80]. Molecular studies confirm its wide distribution across Thailand [81], and a comprehensive survey identified it in 19 of 23 provinces sampled [30]. Two species within the Barbirostris subgroup, *An. hodgkini* (*n* = 2) and *An. pollicaris* (*n* = 23), and one within the Barbirostris complex, *An. donaldi* (*n* = 32), were molecularly identified for the first time in Surat Thani Province, suggesting prior misidentification based solely on morphology [51]. *An. wejchoochotei* was the predominant species of the complex in recent surveys across numerous provinces, including Ayutthaya, Kanchanaburi, Ratchaburi, Phetchaburi, Nan, Chanthaburi, Chiang Mai, Chaiyaphum, Chumphon, Kamphaeng Phet, Khon Kaen, Mahasarakham, Mukdahan, Prachuap Khiri Khan, Sa Kaeo, and Udon Thani [41, 78, 82], and in Ranong Province [37] (Fig. 3A).

Within the Barbirostris complex, *An. saeungae* was reported from Lampang, Phetchaburi, Ratchaburi, Sa Kaeo, Trat, Ubon Ratchathani, and Ranong provinces [37, 41] (Fig. 3A). *An. dissidens* occurred in western and southern Thailand [30], mostly in mountainous areas, with distributions reported in Chiang Mai, Mae Hong Son, Sa Kaeo, Tak, and Trat provinces [41]. *An. campestris* and *An. barbirostris* were reported as rare in recent studies [41].

#### *Anopheles* (Cellia) *sundaicus* (Rodenwaldt) species complex (Sundaicus complex)

As a member of the *An. sundaicus* complex [83], *An. epiroticus* is a malaria vector occurring along the coastline of Southeast Asia. Its immature stages develop in habitats with varying salinity, from low and brackish to seawater concentrations [84], and it is widely distributed throughout eastern and southern Thailand [85]. A recent study in Ranong Province, a region highly endemic for *P. knowlesi* [86], identified *An. epiroticus* as a dominant species, representing 19.3% of *Anopheles* females collected [37]. In a separate study on Chang Island in Trat Province (eastern Thailand), *An. epiroticus* was also the most dominant coastal species and a likely important vector [87]. Owing to its anthropophilic and exophagic behavior [17], this species is considered a secondary vector in eastern Thailand [1].

#### *Anopheles hyrcanus* group

The *An. hyrcanus* group in Thailand comprises two subgroups, Lesteri and Nigerrimus [88], and at least eight species: *An. argyropus*, *An. crawfordi*, *An. nigerrimus*, *An. nitidus*, *An. lesteri*, *An. peditaeniatus*, *An. pursati*, and *An. sinensis* [89, 90]. A multiplex PCR assay has been developed to identify eight Thai *Hyrcanus* group species accurately; among these, *An. nigerrimus*, *An. peditaeniatus*, and *An. sinensis* are suspected vectors of *P. vivax* [89]. Furthermore, *An. peditaeniatus* has been identified as a potential vector for the goat malaria parasite, *P. bubalis* type I, in Thailand [91]. The distribution of molecularly confirmed *Hyrcanus* group sibling species is shown in Fig. 3B.

Studies on the *An. hyrcanus* group in Thailand reveal significant cytogenetic diversity. *An. nitidus* exhibits five metaphase karyotypes, designated as form A (X1, Y1), form B (X1, Y2), form C (X2, Y3), form D (X1, X3, Y4), and form E (X1, X2, X3, Y5) based on sex chromosome variations [92]. Forms D and E were localized in Ubon Ratchathani Province, while forms A, B, and C were found in Phang Nga Province [92]. Similarly, *An. crawfordi* demonstrated four metaphase karyotypes, of which forms A (X1, X2, X3, Y1) and B (X1, X2, X3, Y2) occur in both Thailand and Cambodia, whereas forms C (X2, Y3) and D (X2, Y4) are restricted to Trang Province [93] (Fig. 3B). In Khon Kaen Province, *An. nigerrimus* was used as an outgroup species for a phylogenetic comparison of the Barbirostris complex [41]. The *Hyrcanus* group is notably predominant along the Thai–Lao border in Ubon Ratchathani Province [73]. Within this group, *An. peditaeniatus* is the most widely distributed species in Thailand and was most common in Ubon Ratchathani [3]. Other members, including *An. nigerrimus*, *An. nitidus*, *An. argyropus*, and *An. sinensis*, were also present at varying densities across villages in the same area [3]. *An. peditaeniatus* and *An. pursati* have been recorded in several provinces, including Kanchanaburi, Ratchaburi, Phetchaburi, and Nan [82]. Seasonal abundance of the *Hyrcanus* group is typically highest during the rainy season [73].

#### *Anopheles subpictus* group

From 2013 to 2025, surveys across Thailand identified members of the *An. subpictus* complex [61, 94], which is widely distributed, including northwestern Thailand [58] and Surat Thani Province in the south [51]. These mosquitoes are often found near livestock; for example, one study reported greater abundance on cows than on humans along the Thai–Myanmar border [72]. In Ratchaburi Province, *An. subpictus* s.l. occurred at higher abundance than *An. barbirostris* s.l. [95], and it was collected at goat farms in Kanchanaburi and Ratchaburi provinces in sympatry with *An. vagus* [82]. Recent field collections confirmed *An. subpictus* in Mae Sot District (Tak Province) and Sop Moei District (Mae Hong Son Province) [58] and in Surat Thani Province, southern Thailand [51].

### Role of vectors in human and zoonotic malaria transmission

In Southeast Asia, *P. knowlesi* is the most widely recognized and prevalent zoonotic malaria parasite infecting humans [14, 96–99]. Sporadic human infections with other simian *Plasmodium* species have also been reported, including *P. cynomolgi* in neighboring Cambodia [100] and in Thailand [101], and mixed infections involving *P. inui* and *P. fieldi* in Thailand [102]. These zoonotic malarias are primarily transmitted by mosquitoes of the Leucosphyrus group, which serve as bridge vectors between simian reservoir hosts (primarily macaques) and humans [103].

The Leucosphyrus group comprises key vectors for *P. knowlesi* transmission in Southeast Asia, including *An. dirus* and *An. cracens* of the Dirus complex, and *An. latens*, *An. introlatus*, and *An. balabacensis* of the Leucosphyrus complex [77, 103]. *An. latens* and *An. introlatus* were identified as natural vectors on the basis of detection of NHP *P. knowlesi* DNA and implicated in zoonotic transmission in southern Thailand [77]. Further research implicated *An. latens* in natural transmission of *P. inui* and *P. fieldi*. In southern Thailand, *An. introlatus* has been identified as a vector for *P. cynomolgi* and *P. inui*, and a potential vector for *P. hylobati*, as DNA of these parasites was detected in its salivary glands [77] (Table 1).

**Table 1:** Infectivity of anthropophilic and simiophilic *Anopheles* species reported in Thailand, 2013–2024

Reference	Location/province	*Plasmodium* species	Infectivity rate	Parasite infection group	Parasite detection method	*Anopheles* mosquito	Habitat
[29]	Pak Tho District, Ratchaburi Province	*P. vivax*	0.4%	Human	Single-round multiplex PCR	*An. minimus*	Village
[58]	Mae Sot District, Tak Province	*P. vivax*	0.1%	Human	Real-time PCR	*An. minimus*	Rubber, fruit, and agricultural crop lands
[78]	Thasongyang District, Tak Province	*P. vivax*	0.7%	Human	ELISA	*An. annularis* s.l	Village
[78]	Thasongyang District, Tak Province	*P. vivax*	1.7%	Human	ELISA	*An. barbirostris* s.l	Village
[173]	Bo Rai District, Tart Province	*P. vivax*	2.8%	Human	Nested PCR	*An. dirus*	Rubber plantation
[78, 172]	Thasongyang District, Tak Province	*P. vivax*	0.4%	Human	ELISA	*An. maculatus* s.l	Village
[78, 172]	Thasongyang District, Tak Province	*P. vivax*	0.8%	Human	ELISA	*An. minimus* s.l	Village
[173]	Thong Pha Phum District, Kanchanaburi Province	*P. vivax*	28.8%	Human	Nested PCR	*An. aconitus*	Near rubber plantation
[46]	Na Chaluai District, Ubon Ratchathani	*P. vivax*	3.5%	Human	ELISA and nested PCR	*An. dirus*	Wood hut near national park
[46]	Na Chaluai District, Ubon Ratchathani	*P. vivax*	1.0%	Human	ELISA	*An. philippinensis*	Wood hut near national park
[69]	Thasongyang District, Tak Province	*P. vivax*	0.1%	Human	ELISA	*An. maculatus*	Village
[174]	Mae Sot, Tak Province5	*P. vivax*	0.5%	Human	PCR	*An. minimus*	Village
[91]	Chachoengsao Province	*P. bubalis*	5.7%	Buffalo	PCR and sequsencing	*An. campestris* or *An. wejchoochotei*	Buffalo farm
[91]	Chachoengsao Province	*P. bubalis*	2.5%	Buffalo	PCR and sequencing	*An. peditaeniatus*	Buffalo farm
[124]	Not specify	*P. caprae*	1.4%	Goat	PCR and sequencing	*An. aconitus*	Goat farm
[77]	Narathiwat Province, Southern Thailand	*P. cynomolgi*	2.8%	Simian	Species-specific PCR and Sequencing	*An. introlatus*	Village
		*P. hylobati*	2.8%				
		*P. knowlesi*	2.8%				
		*P. inui,*	8.1%				
		*P.* spp.	2.8%				
[77]	Narathiwat Province, Southern Thailand	*P. knowlesi*	1.5%	Simian	Species-specific PCR and sequencing	*An. latens*	Village
		*P. inui*	1.5%				
		*P. fieldi*	3.1%				
		*P. coatneyi*	1.5%				
		*P. hylobati*	1.5%				
		*P. juxtanucleare*	1.5%				
		*P.* spp.	6.2%				
[77]	Narathiwat Province, Southern Thailand	*P.* spp.	50.0%	*—*	Sequencing	*An. macarthuri*	Village
[77]	Narathiwat Province, Southern Thailand	*P. caprae*	0.7%	Goat	Species-specific PCR	*An. maculatus*	Village
[124]	Not specified	*P. caprae*	0.9%	Goat	PCR and sequencing	*An. subpictus*	Goat farm
[7]	Kaeng Khoi District, Saraburi	*P. cynomolgi*	20.0%	Simian	Ultrasensitive ddPCR1	*An. dirus*	Forest
[7]	Kaeng Khoi District, Saraburi	*P. cynomolgi*	6.3%	Simian	Ultrasensitive ddPCR1	*An. minimus*	Forest
[7]	Kaeng Khoi District, Saraburi	*P. cynomolgi*	21.8%	Simian	Ultrasensitive ddPCR1	*An. sawadwongporni*	Forest

The major human malaria vector *An. dirus* is widespread in Thailand (Fig. 2) and transmits human as well as NHP malaria parasites. Its adaptability to anthropophilic, simiophilic, and zoophilic hosts may contribute to escalating *P. knowlesi* incidence by bridging infection between monkeys and humans. *An. maculatus* has recently been identified as a potential vector for *P. knowlesi* after experimental infection in Thailand [103] and for the ungulate malaria parasite *P. caprae* in southern Thailand [77] (Table 1).

A recent study of the vectorial capacity of *An. dirus* to acquire *P. knowlesi* from humans demonstrated a human-to-mosquito transmission cycle under laboratory conditions [103]. This finding highlights the risk of a potential sustained human–mosquito–human transmission cycle in nature. Vector feeding preferences and habitats are crucial to zoonotic transmission, particularly because *P. knowlesi* occurs where macaque reservoirs are present and forest-related activities increase risk [104]. Human activities such as hunting or logging in forest-fringe areas increase exposure to vectors and facilitate contact among humans, infected monkeys, and mosquitoes [105].

Investigation of local vector ecology is essential because vector behavior varies regionally; a malaria vector in one area may not be a vector elsewhere [37]. Local studies are needed to identify the specific *Anopheles* species transmitting *P. knowlesi* in each location, and to monitor behavioral changes, including potential host shifts from macaques to humans, to understand transmission dynamics [103].

New molecular and epidemiological approaches are needed to track the complex transmission cycles of NHP malaria, which involve spillover from NHP to humans and spillback to wild NHP populations [106]. According to Masse et al. (2025), a significant knowledge gap concerns human–vector overlap and behaviors that drive zoonotic malaria transmission [107]. Limited understanding of how human activities, such as forest-related work, alter vector biting and resting behaviors hampers prediction of when and where humans are most at risk. Moreover, the lack of integrated surveillance that simultaneously monitors human infection, NHP parasite prevalence, and vector abundance and behavior leads to data fragmentation, complicating accurate modeling of transmission and the implementation of targeted interventions. This gap underscores the need for a One Health approach that integrates entomological, epidemiological, and ecological research to understand these interactions. Environmental change increases interactions between humans and wildlife, and it remains unclear how future environmental and anthropogenic changes might elevate NHP malaria risk [106].

### *Anopheles* bionomics

#### Life history traits

External stressors can significantly influence the survival of *An. minimus* populations [44]. Higher temperatures, within an optimal range, increase vectorial capacity by accelerating parasite development and prolonging mosquito longevity [108]. This enhanced vectorial capacity, in turn, contributes to greater complexity in mosquito ecology and leads to heightened malaria transmission [15].

Intraspecific variability in host feeding varies among populations of *An. dirus* in Thailand and across the GMS and is likely influenced by environmental factors and host availability [15, 109]. Feeding on sheep blood significantly reduced the survival of *An. dirus*, *An. cracens*, and *An. minimus* compared with human, chicken, or mouse blood [110]. This negative effect extended to the next generation: offspring of *An. dirus* and *An. cracens* whose parents fed on sheep blood had significantly shorter lifespans [110]. A shorter lifespan lowers the entomological inoculation rate and reduces progeny fitness, suggesting that the presence of certain nonhuman hosts can act as a dead end for vector populations and may naturally reduce transmission risk to humans.

A study of *An. dirus* experimentally infected with *P. knowlesi* detected oocysts in midguts at day 7 and sporozoites in salivary glands at day 14 post-infection, demonstrating competence to transmit *P. knowlesi* [103]. This is notable because *An. dirus* is a confirmed zoonotic vector in Vietnam [15, 111, 112] and occurs where *P. knowlesi* is an increasing concern. These results reinforce the need to investigate the role of *An. dirus* in *P. knowlesi* transmission in Thailand.

A blood meal triggers physiological changes in *An. dirus* females, including upregulation of genes involved in oogenesis [113], confirming the link between blood feeding and reproductive capacity. *Anopheles* mosquitoes, including *An. dirus*, commonly take a first blood meal before mating and use ingested nutrients to support egg development [113]. This behavior supports oogenesis and contrasts with autogeny, in which eggs develop without a blood meal [114]. Using a forced oviposition technique, overall oviposition rates were similar between laboratory strains (36.7%) and field populations (36.9%), although species-level variation was marked. Success rates for laboratory-reared *An. dirus* and *An. cracens* were 66.0% and 12.0%, respectively. In contrast, only 8.0% of wild-caught *An. dirus* females from Pu Teuy Village, Sai Yok District, Kanchanaburi Province produced eggs [115]. The mean number of eggs per female induced by forced oviposition was higher for laboratory-reared *An. dirus* (93.61 ± 46.37) than for *An. cracens* (39.33 ± 17.75), and differed from field populations of *An. harrisoni* (36.34 ± 38.22) and *An. barbirostris* s.l. (147.58 ± 85.07) [115]. This reproductive performance indicates biological plasticity and suggests that colony females can retain eggs and, when prompted by constraints such as a lack of suitable sites or a physiological trigger from the forced technique, deposit an entire batch. Although this may not reflect natural behavior, it demonstrates high reproductive potential, a key factor in vector effectiveness.

#### Breeding habitat selection

Breeding-site preference is a critical determinant of mosquito distribution. Favorable habitats for anopheline larvae include permanent water bodies with aquatic vegetation and shaded areas, such as those near marginal streams [105]. Since 2013, research has uncovered diverse and often cryptic breeding-site preferences for *Anopheles* vectors in Thailand. For instance, *An. dirus*, typically associated with forested habitats, was found breeding in an unusual location, namely a dark limestone cave in western Thailand [116]. Another study identified larvae in stagnant water seeping from grooves in a large rock within shaded primary forest [52]. The highly cryptic and dispersed nature of *An. dirus* breeding sites poses a significant challenge for larval source management because they do not meet WHO’s “fixed, few, and findable” criteria for effective vector control [117]. One survival strategy employed by the *An. dirus* complex mosquitoes during the dry season involves the exploitation of cryptic habitats, including manmade structures such as underground water wells in Chanthaburi Province [118]. This adaptation underscores the difficulty of controlling *An. dirus* populations, as traditional larval source management often targets more visible sites and can miss these hidden reservoirs.

The discovery of *An. baimaii* in Koh Kood District, Trat Province, near Cambodia, which is the easternmost occurrence of this species in Thailand, challenges earlier records that confined it to central, southern, and western forest regions, particularly along the Thai–Myanmar border [45, 55]. Typical breeding habitats of *An. baimaii* are small, temporary forest-fringe water bodies such as ditches, elephant footprints, cattle hoof marks, and wheel tracks [119]. Larvae of *An. minimus* have been observed in unusual sites, namely crab holes and swamps in Doi Inthanon National Park, Chiang Mai Province [120], as well as a domestic water tank in suburban Hanoi, Vietnam [121]. These findings demonstrate adaptability beyond its more typical streambed habitats.

In Samut Songkhram Province, the coastal vector *An. epiroticus* showed a significant larval preference for brackish water containing green algae [122]. Entomological surveys in southern Thailand identified breeding habitats of forest-dwelling Leucosphyrus complex species, including *An. introlatus* and *An. latens*, with larvae found in ground pools, animal footprints, wheel tracks, and small, shallow running streams [77]. This baseline entomological information is critical for understanding simian malaria transmission and for developing control strategies.

#### Feeding behavior

Recent studies provide detailed insights into host preference and selection patterns of *Anopheles* mosquitoes, which are shaped by species-specific bionomics and environmental factors. Many *Anopheles* species in Thailand show a strong preference for animal hosts, such as cattle, over humans; this zoophily significantly impacts transmission dynamics [79, 123]. Buffalo bait is a potent attractant for species such as *An. epiroticus* in coastal regions of Thailand [87]. Most species of the *An. hyrcanus* group are highly zoophilic and feed on cattle. Along the Thai–Myanmar border, *An. peditaeniatus* showed a high preference for cattle over humans (zoophilic index 98.7%, *n* = 7345) and is therefore considered highly zoophilic [3]. *An. dirus*, a primary malaria vector, is characterized as an exophilic, anthropophagic feeder, with a tendency to feed and rest outdoors, particularly where indoor residual spraying (IRS) is used, which drives host seeking outdoors [11]. Depending on location and environment, *An. minimus* is often exophilic in Thailand, with a high exophagic rate and preferential outdoor feeding [73]. In other countries, such as Vietnam, this species can exhibit both anthropophilic and zoophilic tendencies, as well as endophagic behavior [39, 109]. *An. minimus* displayed strong zoophagy on the Thai–Myanmar border, with ratios of 77.0% and 87.0% [123]. Its peak biting activity typically occurs from early evening until midnight. *An. harrisoni* is primarily zoophilic, preferring animal hosts, whereas the closely related sibling *An. minimus* is an opportunistic feeder that bites both humans and animals [66].

Mosquitoes in the Maculatus group, specifically *An. maculatus*, exhibited strong zoophagy and a high zoophilic index [72, 123]. Within this group, *An. sawadwongporni* and *An. pseudowillmori* also showed high zoophilic indices, indicating a preference for animal hosts [72]. Similarly, species within the Barbirostris complex such as *An. dissidens* and *An. saeungae* are mainly zoophilic [30], in contrast to the more anthropophilic *An. wejchoochotei* and *An. campestris* [61].

During the review period, 60.7% (*n* = 49,569) of primary vectors were identified using HLC compared with 0.8% (*n* = 136) using cattle-baited traps (CBT) (Table 2). For secondary vectors, HLC and CBT recorded 24.4% (*n* = 4,784) and 20.5% (*n* = 4,021), respectively (Table 3).

**Table 2:** Abundance of primary *Anopheles* malaria vectors by collection method

Collection method	*An. dirus* s.l.	*An. dirus* s.s.*	*An. minimus* s.l.	*An. minimus* s.s.*	*An. maculatus* s.l.	An. maculatus*	Total collection by collection method	Reference
BG-Pro CDC-style traps	308	66	208	48	254	27	911	[37, 40, 41, 45, 148]
BGS and CDC LT	0	0	1581	0	0	0	1,581	[142]
CBT only	1	5	35	8	66	21	136	[79, 181]
CDC light trap	81	43	8045	222	2806	16	11,213	[7, 44, 66, 69, 78, 85, 91, 94, 105, 124, 147, 172, 175–178]
HLC only	1478	366	40215	35	7447	28	49,569	[46, 51, 52, 77, 81, 153, 174]
HLC, HDT, and HDNT	0	2	0	419	0	3	424	[141]
HLC and CBT	15	323	174	13666	139	3309	17,626	[29, 30, 47, 58, 72, 76, 87, 169]
UV, LED, and fluorescent	0	4	0	5	0	0	9	[143, 144, 179]
Not specified	49	0	37	0	14	0	100	[173]

*CBT* cattle baited trap, *HLC* human landing catch, *HDNT* human double net trap, *HDT* human-baited host decoy trap, *CDC LT* CDC light trap

^*^Confirmed by molecular identification technique

**Table 3:** Abundance of secondary and suspected *Anopheles* malaria vectors by collection method

Collection method	*An. aconitus*^1^	*An. aconitus*^1*^	*An. pseudaowillmori*^1^	*An. pseudaowillmori*^1*^	*An. sawadwongoni*^2^	*An. sawadwongoni*^2*^	*An. barbirostris* s.l.^2^	*An. barbirostris* s.s.^2*^	*An. campestris*^2^	*An. campestris*^2*^	*An. philippinensis*^2^	*An. philippinensis*^2*^	Total collection by collection method	Reference
BG, Mosquito Magnet, CDC UV, CDC backpack	0	0	0	0	0	0	160	0	0	0	0	0	160	[180]
BG-Pro CDC-style traps	0	23	0	2	200	7	308	0	0	0	9	4	553	[37, 40, 41, 45, 148]
BGS and CDC LT	0	0	0	0	162	0	0	0	0	0	0	0	162	[142]
Black light trap and Kelambu Trap	0	0	0	0	0	0	0	0	0	153	0	0	153	[61]
CBT only	0	2	0	0	0	16	3497	0	0	0	479	27	4021	[79, 164]
CDC light trap	733	0	73	11	339	183	1339	0	51	0	18	0	2747	[7, 44, 66, 69, 78, 85, 91, 94, 105, 124, 147, 172, 175–178]
HLC only	3	2	0	35	0	19	4695	0	0	19	11	0	4784	[51, 52, 77, 81, 153, 174]
HLC and CBT	28	286	0	160	0	989	3887	3	1	0	1325	144	6823	[29, 30, 46, 47, 58, 72, 73, 76, 87, 169]
Mosquito magnet	0	0	0	0	0	0	149	0	0	0	0	0	149	[145, 182]
UV, LED, and fluorescent	0	1	0	0	0	8	4	0	0	0	17	0	30	[143, 144, 179]
Not specified	0	7	0	0	0	0	0	0	0	0	0	0	7	[173]

*CBT* cattle baited trap, *HLC* human landing catch, *HDNT* human double net trap, *CDC LT* CDC light trap

Multiple *Anopheles* species, including the *An. minimus*, *An. dirus*, and *An. barbirostris* complexes; the Maculatus group; the *An. hyrcanus* group (notably *An. peditaeniatus*); the Annularis group (*An. annularis*, *An. nivipes*, and *An. philippinensis*); and species such as *An. aconitus*, *An. epiroticus*, and *An. subpictus*, have been frequently collected using animal-baited traps (Tables 2 and 3). Collections at buffalo farms in Chachoengsao Province yielded *An. wejchoochotei*, *An. campestris*, and *An. peditaeniatus*, species implicated as vectors for *P. bubalis* (Table 1) [91]. Surveys at goat farms using aspirators or light traps in Kanchanaburi, Nan, Ratchaburi, and Phetchaburi identified probable vectors for *P. caprae* [124]. Further investigations using blood-meal PCR assays are warranted to determine the anthropophilic, zoophilic, or zoo-anthropophilic origins and preferences of *Anopheles* vectors [37].

Geographic variation in feeding behavior among primary vectors was evident in paired HLC and CBT collections between distant locations in Tak and Trat provinces separated by 600 km, and also within smaller areas in Tak Province (Table 4). *An. aconitus* was an exception, showing consistent zoophagy in Tak. Such differences can produce distinct epidemiological situations and influence the effectiveness of control measures. Although deploying both animal-baited traps (ABT) and HLC at the same site would provide a more complete picture of vector behavior, animal-baited traps are rarely used owing to logistical challenges [125]. As a complement to HLC, animal-baited traps could broaden the range of observed behaviors, in line with WHO recommendations for a wider array of surveillance methods [125, 126]. Expanding entomological data from both animal-baited traps and HLC is crucial for a comprehensive understanding of vector behavior.

**Table 4:** Proportion of *Anopheles* mosquitoes collected from paired outdoor cattle bait trap (CBT) and human landing catch (HLC) at three sites in Thailand

Province (Ref.)	Proportion in percentage and (numbers) of mosquitoes caught in								Total
	Primary vectors			Secondary vectors			Other *Anopheles*		
	Species	CBT	HLC^§^	Species	CBT	HLC^§^	CBT	HLC^§^	
Trat [87]	*An. dirus*	0.15 (*n* = 8)	2.44 (*n* = 132)	–			82.29 (*n* = 4443)	14.15 (*n* = 764)	5399
	*An. minimus*	–	0.16 (*n* = 9)	–					
	*An. maculatus*	0.80 (*n* = 43)		–					
Tak [72]	*An. dirus*	0.02 (*n* = 1)	0.70 (*n* = 30)	*An. aconitus*	0.65. (*n* = 28)	0.07 (*n* = 3)	33.73 (*n* = 1451)	3.74 (*n* = 161)	4301
	*An. minimus*	9.70 (*n* = 417)	33.85 (*n* = 1,456)	–					
	*An. maculatus*	10.49. (*n* = 451)	7.04 (*n* = 303)	–					
Tak [47]	*An. dirus*	0.05 (*n* = 9)	0.10 (*n* = 17)	*An. aconitus*	1.32 (*n* = 218)	0.09 (*n* = 15)	32.38 (*n* = 5333)	1.07 (*n* = 176)	16,468
	*An. minimus*	38.68 (*n* = 6369)	14.86 (*n* = 2447)	*An. sawadwongporni*	0.98 (*n* = 161)	0.04 (*n* = 6)			
	*An. maculatus*	10.00 (*n* = 1647)	0.38 (*n* = 62)	*An. pseudowillmori*	0.04 (*n* = 7)				
			–	*An. baimaii*	0.00 (*n* = 1)				
Prachuap Khiri Khan [75]*		0	0		0	0	25.12 (*n* = 52)	74.88 (*n* = 155)	207

^*^Only Barbirostris complex species have been reported

^§^Conducted indoors and outdoors

Although mosquito surveillance and distribution studies exist for Thailand, monkey-baited traps are more commonly reported in Malaysia [127–133], indicating a knowledge gap in Thailand regarding mosquito species that feed on nonhuman primates in natural settings. This limits understanding of zoonotic malaria transmission dynamics in Thailand and needs to be addressed.

**Fig. 4:**
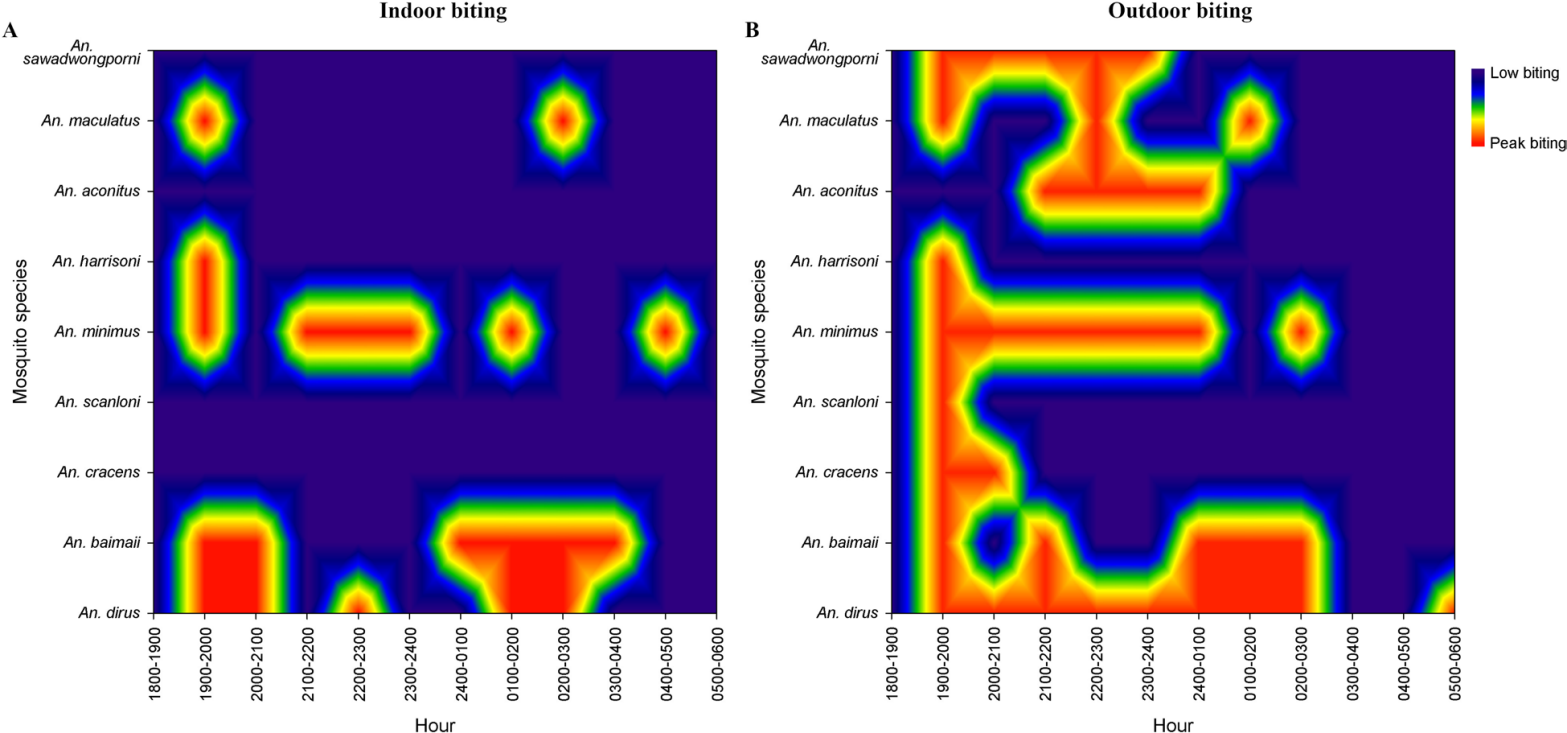
Peak biting patterns of molecularly confirmed major malaria vectors in indoor (**A**) and outdoor (**B**) habitats. Low biting indicates less than a 0.1 biting rate (bites per person per night), and peak biting indicates a biting rate greater than or equal to 1.0 biting rate (bites per person per night)

Among *Anopheles* species confirmed by molecular techniques, only *An. dirus*, *An. baimaii*, *An. minimus*, *An. harrisoni*, and *An. maculatus* showed indoor biting activity (Fig. 4A). *An. dirus* and *An. baimaii* exhibited the highest indoor biting in the early evening (1900–2100) and again from midnight to 0400. *An. dirus* biting patterns show marked geographic variation: in Tak Province where most indoor mosquitoes were caught between 2200–2300 and 0400–0500, with outdoor peaks at 2100–2200 and 2300–0000 [123]. In Ubon Ratchathani, biting occurred indoors and outdoors from 2000–2300 and again from 0100–0300 [73]. On Chang Island, Trat Province, the indoor peak was 0000–0100, while the outdoor peak occurred earlier, 1900–2300 [87].

In areas where IRS was used, indoor biting peaks shifted toward earlier or outdoor biting [109]. While the outdoor peak remained unchanged, a higher proportion of mosquitoes began biting earlier in the evening, suggesting that IRS altered behavior and pushed mosquitoes to seek blood meals outdoors or earlier. In contrast, *An. minimus* exhibited a consistent peak in indoor biting throughout the night (Fig. 4A). DDT use had no detectable effect on the already early-biting *An. minimus* s.l. population [134]. However, widespread IRS altered *An. minimus* s.l. behavior [135], likely reflecting a species shift from *An. minimus* to *An. harrisoni*, a phenomenon also observed in Vietnam with extensive use of insecticide-treated nets [39]. Although *An. harrisoni* showed early evening indoor peaks (1900–2000) and another after midnight, a causal link with insecticide-treated nets has not been established. The bimodal biting of *An. maculatus*, peaking at 1900–2000 and again at 0200–0300, is well documented.

*An. minimus* biting patterns vary geographically. In rubber plantations in Trat Province, peaks occurred at 1900–2100 and 0100–0300 [11]. In Ubon Ratchathani, indoor biting was highest at 1900–2100, while outdoor peaks were 1900–2000 and 2300–0000 [73]. In farm huts in Tak Province, activity began as early as 1700–1800, peaked at 2000–2100, decreased in the early morning, then resurged from 0300 and remained relatively high until 0600–0700 [123].

In Thailand, *An. maculatus* outdoor biting also varies geographically. In Tak and Surat Thani provinces, peak outdoor activity occurred at 1900–2000 [51, 58, 123]. In Narathiwat, abundance peaked at 2100–2300 and declined after midnight [77]. Indoor biting generally peaked at 2100–2200, with most indoor biting occurring before 2200 [123]. Early morning activity was also reported, with some bites after 0500 [123] (Fig. 4B).

Given the early biting times of *Anopheles* vectors, the first human behavior observations on the Thai–Myanmar border demonstrated the role of key drivers and modulators in sustaining infection prevalence [123]. By integrating human behavior observations with mosquito collections in villages and forested farm huts used for subsistence farming, exposure was shown to occur predominantly outdoors, particularly among nonusers of long-lasting insecticidal nets. Risk was exacerbated by suboptimal bed net coverage. Early waking hours, when people had left the safety of their nets, coincided with later morning biting peaks. This adaptive mosquito behavior, an evolutionary response to indoor insecticidal interventions such as insecticide-treated nets, enables mosquitoes to obtain a blood meal before people are under nets, thereby avoiding insecticide contact [69]. This is a major driver of residual transmission, where malaria persists despite high coverage of conventional control measures.

#### Infection rates and *Plasmodium* species in *Anopheles* mosquitoes

**Fig. 5:**
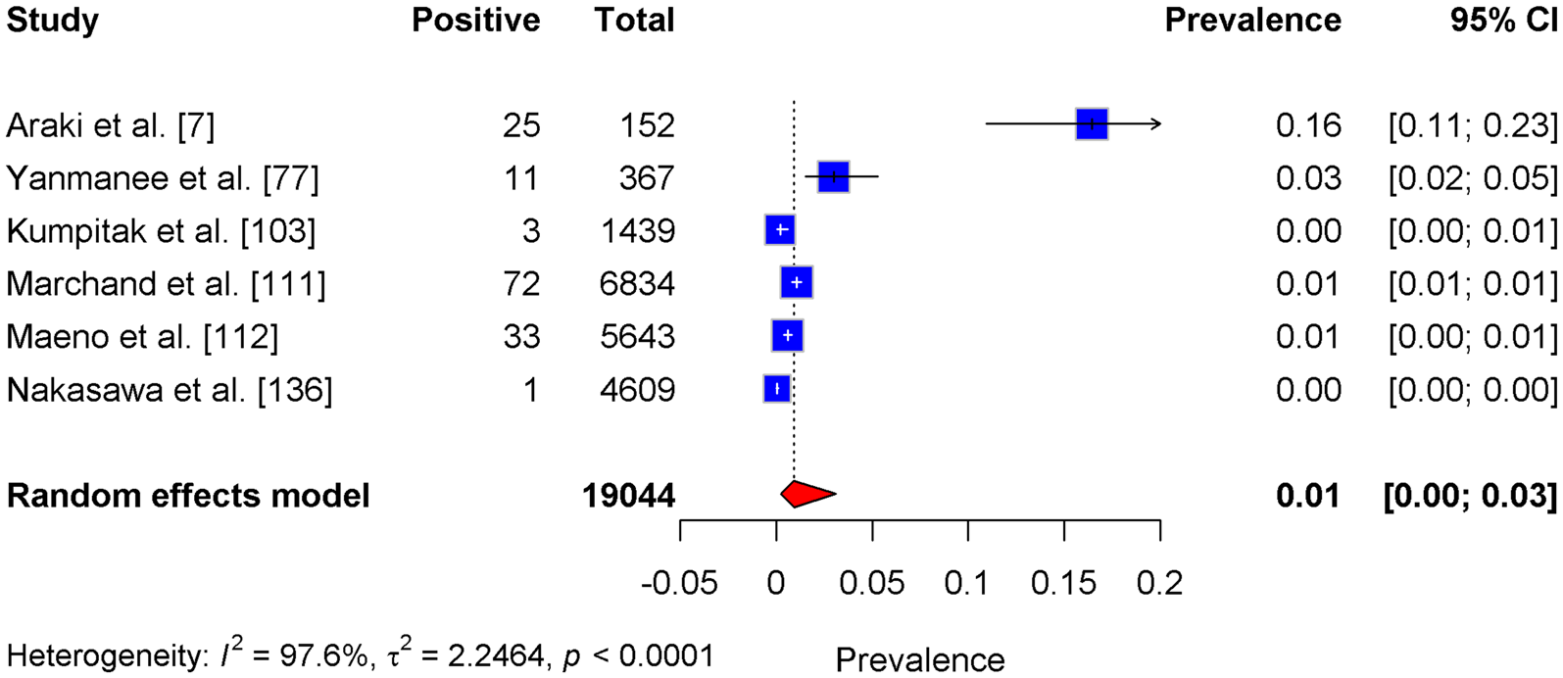
Forest plot of the prevalence of nonhuman primate *Plasmodium* infection in *Anopheles* mosquitoes in Vietnam [111, 112, 136] and Thailand [7, 77, 103]. Individual study estimates and pooled prevalence values (random-effects model) are shown for five *Anopheles* species across three studies from Vietnam and three studies from Thailand. Error bars represent 95.0% confidence intervals (CI). *ES* estimated prevalence

Six studies from Thailand (*n* = 3) and Vietnam (*n* = 3) reporting *Plasmodium* infections in *Anopheles* mosquitoes were included in the meta-analysis (Fig. 5; Table 5). Overall mosquito infection prevalence was low (pooled prevalence = 0.01; 95% CI 0.00–0.03), but estimates varied markedly between settings, with substantial heterogeneity (*I*^2^ = 97.6%, *P* < 0.0001). The highest infection prevalence (0.2%) among 152 mosquitoes from six anopheline species in Thailand was attributed to a droplet digital PCR (ddPCR), a highly sensitive PCR method as shown in Fig. 5 [7]. In Vietnam, *An. dirus* showed a very low pooled prevalence (0.02%; 95% CI: 0.00–0.00%), consistent with sporadic detection of infected mosquitoes, with the lowest estimate in Khanh Hoa Province (*n* = 1; pooled prevalence = 0.00) [136]. Nevertheless, molecular evidence of co-infection with *P. vivax, P. falciparum* and *P. knowlesi* in a single wild-caught *An. dirus* indicates that vectors in these settings can harbor both human and zoonotic malaria parasites simultaneously [136], underscoring the potential for cross-species transmission even where overall infection prevalence is low. These findings suggest that low average infection prevalence may mask focal transmission hotspots where ecological conditions favor intense enzootic circulation and increased human exposure.

**Table 5:** Prevalence of *Plasmodium* infection and parasite species detected in *Anopheles* mosquitoes included in the meta-analysis

No	Reference	Country	Province	Mosquito infectivity (%)	Year(s) of study	Number of mosquitoes with *Plasmodium* species identified by PCR		*Anopheles* species incriminated
						Pk	Others	
1	[136]	Vietnam	Khanh Hoa	0.02	2007–2008	1	Pf (*n* = 1), Pv (*n* = 1), Pm (*n* = 1)	*An. dirus*
2	[111]	Vietnam	Khanh Hoa	1.00	2008–2010	31	Pf(*n* = 36), Pv (*n* = 36), Pm (*n* = 4)	*An. dirus*
3	[112]	Vietnam	Khanh Hoa	0.59	2008–2010	33	Pf (*n* = 33), Pv (*n* = 35), Pm (*n* = 3)	*An. dirus*
4	[77]	Thailand	Narathiwat	2.99	2018–2019	2	Pin (*n* = 6), Pcy (*n* = 1), Pfi (*n* = 2), Pco (*n* = 1)	*An. latens*, *An. introlatus*
5	[103]	Thailand	Yala	0.21	2023	3	-	*An. dirus*
6	[7]	Thailand	Saraburi	16.44	2023	-	Pcy (*n* = 25)	*An. sawadwongporni*, *An. minimus*, *An. dirus*

Data are summarized by country, province, study period, mosquito infection prevalence, *Anopheles* species incriminated, and *Plasmodium* species identified by PCR. Studies from Vietnam are presented as comparators

*Pf* *P. falciparum,* *Pv* *P. vivax*, *Pk* *P. knowlesi*, *Pcy* *P. cynomolgi*, *Pm* *P. malariae*, *Pin* *P. inui*, *Pfi* *P. fieldi*, *Pco* *P. coatneyi*

In contrast, substantially higher infection prevalences were reported in Thailand, particularly in Saraburi Province, where *P. cynomolgi* infection reached 16.4% (*n* = 25) in *An. sawadwongporni* (*n* = 17), *An. minimus* (*n* = 5), and *An. dirus* (*n* = 3) [7]. This finding represents the first detection of simian malaria parasite DNA in wild-caught mosquitoes with infection rates of 11.2%, 3.3%, and 2.0% in *An. sawadwongporni*, *An. minimus*, and *An. dirus*, respectively [7], and highlights the capacity of multiple vector species to sustain NHP malaria transmission. Additional studies from southern Thailand in Narathiwat Province documented diverse NHP *Plasmodium* species, including *P. knowlesi* (*n* = 2), *P. cynomolgi* (*n* = 1), *P. inui* (*n* = 6), *P. fieldi* (*n* = 2), and *P. coatneyi* (*n* = 1) within *Anopheles* populations, reflecting complex enzootic transmission cycles [77]. Using a random-effects model, the pooled mosquito infection prevalence across Vietnam and Thailand was 0.01 (95% CI 0.00–0.03). Between-study heterogeneity was substantial (*I*^2^ = 97.6%, Cochran’s *Q*-test *P* < 0.0001), indicating considerable variability in infection prevalence estimates across studies. The high level of heterogeneity likely reflects differences in vector species composition, ecological context, sampling intensity, and molecular diagnostic approaches, rather than random variation alone. Together, these results reinforce the need for surveillance strategies that extend beyond human case detection to include entomological and reservoir-host monitoring, and they support the application of One Health frameworks to identify and mitigate zoonotic malaria risk in forest-fringe and plantation-dominated landscapes.

Despite a much larger population at risk of malaria (13,606,254 inhabitants; ~ 20.0% of the total population), Thailand has fewer documented vector incrimination studies than Malaysia, which has an extensive body of research on NHP malaria [127–133], although its at-risk population is much smaller (1,405,051 inhabitants). This research gap is a concern for elimination in the GMS, particularly with the rise in indigenous *P. knowlesi* from 22 cases in 2020 to a maximum of 259 cases in 2023 (83 cases in 2024), and the potential emergence of other zoonoses. Limited entomological data on vector incrimination, interactions with humans and monkeys, mosquito longevity, and vector competence hinder understanding of transmission dynamics. A similar challenge is reported in Malaysia, where continued increases in *P. knowlesi* and other NHP cases have delayed malaria certification [137]. Cambodia and Thailand reported 11 and 239 indigenous *P. knowlesi* cases, respectively, in 2023, highlighting a regional challenge [5].

The marked increase in *P. knowlesi* cases in Thailand underscores the need for integrated knowledge of NHP malaria prevalence in humans, macaques, and *Anopheles* vectors [77]. Current entomological studies often rely on dissections to detect parasites and parous rates but lack direct mosquito longevity measurements [116, 138]. Identifying sporozoites in field-collected mosquitoes is also challenging. Our meta-analysis highlights the difficulty of incriminating NHP malaria vectors due to low sporozoite rates. In recent years, only two field studies [7, 77] and one laboratory study [103] have successfully detected NHP malaria parasites (*P. knowlesi*, *P. cynomolgi*, and *P. inui*) in mosquitoes (Table 1).

Secondary and suspected vectors in Thailand, such as *An. aconitus*, *An. epiroticus*, and members of the *An. barbirostris* complex, including *An. campestris* and *An. wejchoochotei,*, were infected with *Plasmodium* in both laboratory and field studies [58]. Notably, *An. wejchoochotei* exhibits high anthropophily and is considered a likely vector in Sa Kaeo Province, eastern Thailand [31]. *An. campestris* also shows anthropophily and has been implicated in transmission of *P. falciparum* and *P. vivax* [61]. The combination of these vectors and the mobility of migrant workers, especially along international borders, underscores the need for expanded surveillance and control strategies [61].

According to recent studies, two members of the Leucosphyrus complex, *An. latens* and *An. introlatus*, are native vectors of *P. knowlesi* in Narathiwat Province [77]. They also show potential to transmit other simian and avian *Plasmodium* species [77]. In central Thailand (Phra Phothisat Temple), *An. sawadwongporni* was found to carry *P. cynomolgi* [7], which is notable given the high prevalence of NHP malaria among wild macaques in that region.

In Ranong Province, an area endemic for *P. knowlesi*, three potential vector species, *An. baimaii*, *An. hodgkini*, and *An. epiroticus*, were identified [37]. These species warrant special surveillance for their vector potential in Thailand and elsewhere.

Beyond NHP malaria, vector–parasite interactions in Thailand are expanding. For the first time, *An. subpictus* and *An. aconitus* have been identified as probable vectors of *P. caprae*, a malaria parasite of goats, suggesting an emerging interface beyond human malaria [124]. In addition, *An. maculatus* has been identified as a vector of *P. caprae* in domestic goats in southern Thailand [77].

### Advanced mosquito surveillance and nonmolecular identification

The human-baited double net trap [139, 140] and human-baited host decoy trap [141] are considered as alternatives to the HLC as they can reduce human exposure to infective bites during mosquito surveillance (Tables 2 and 3). Efficacy varies by location: both traps showed promise as substitutes for conventional outdoor HLC in Vietnamese forests, but performance appeared doubtful in Thai forests [141].

The CDC miniature light trap (CDC LT) is a dependable, efficient method for collecting *Anopheles*; effectiveness is enhanced when combined with CO_₂_, an incandescent light, and a chemical lure (BG-lure) [142]. Studies on light sources indicate that traps fit with fluorescent lights and various LEDs are effective for collecting *Anopheles* [143]. Selecting the optimal wavelength maximizes trap performance: a UV fluorescent light was most efficient, followed by UV LED, blue LED, and green LED. In Nakhon Ratchasima Province, a 365-nm LED was the most effective light source overall, capturing 790 mosquitoes (23.7% of 3,339), but *Anopheles* captures were notably low (8 specimens; 0.2%) [144].

The Mosquito Magnet uses a combination of attractants and performs particularly well in coastal areas [145]. It is reliable where *An. epiroticus*, *Culex sitiens*, and *Culex quinquefasciatus* are prevalent. In Malaysia, Mosquito Magnet efficacy for collecting *Anopheles* (vectors of NHP *Plasmodium*) was comparable to HLC, and it provides an ethical, safer alternative [146].

Geometric morphometrics is a rapid, inexpensive method to discriminate species and sibling complexes via wing-venation shape. Its utility has been demonstrated across multiple taxa: within the *An. dirus* complex, geometric morphometrics distinguished *An. dirus* and *An. baimaii* with 92.4% accuracy [104]; within the *An. minimus* complex, *An. minimus* and *An. harrisoni* were separated with 90% accuracy [66]; in the Maculatus group, *An. maculatus*, *An. sawadwongporni*, and *An. pseudowillmori* were differentiated with 93% accuracy [147]; and within the Barbirostris complex, landmark-based geometric morphometrics distinguished *An. dissidens*, *An. saeungae*, and *An. wejchoochotei* with 74.3% accuracy [41]. Variation in accuracy reflects differing interspecific morphological distances. Geometric morphometrics does not replace genetic analysis but is a fast, low-cost alternative when molecular methods are not feasible, particularly in field settings where specimens are intact.

Artificial intelligence is being developed to support surveillance and vector-borne disease management. A recent study used a self-supervised vision transformer (DINOv2) to classify mosquito species from mobile-phone images, enabling rapid, accurate field identification [148]. The approach successfully classified 16 female mosquito species from across Thailand, including vectors of malaria and dengue [148]. Artificial intelligence is also being applied to automated egg counting for population monitoring and program evaluation.

Biomarkers are valuable for assessing human exposure to *Anopheles* bites and pathogen-transmission risk. They support evaluation of human–vector contact and control efficacy, identification of hotspots, and measurement of exposure in low-transmission settings [149–151]. In Thailand, the gSG6-P1 salivary peptide is a key biomarker [152, 153]. High sequence identities have been shown for *An. minimus* (86.9%) and *An. aconitus* (86.9%), and for *An. maculatus* (82.6%) [153]. Antibody intensity correlates positively with *Anopheles* exposure and entomological inoculation rate. For *An. dirus*, identity is lower (47.8%), indicating the need for further research [153].

### New vector control methods

Newer interventions for anopheline control include volatile pyrethroid spatial repellents, such as transfluthrin and metofluthrin. These products create a “vector-free space” through noncontact irritancy, preventing landing, inhibiting feeding, and reducing mosquito–human contact in outdoor or semi-enclosed spaces [140, 154, 155]. Other methods include insecticide-treated clothing with an active ingredient such as etofenprox, which provides personal protection via contact irritancy and requires mosquitoes to land on treated fabric [140]. Topical repellents such as picaridin form a surface barrier against bites. These products are evaluated for their capacity to disrupt feeding cycles, thereby reducing vector competence and lowering disease risk [154].

Mass drug administration of ivermectin in humans or livestock is being explored as a tool for malaria control and elimination. Ivermectin is lethal to multiple *Anopheles* species, including *An. minimus*, *An. campestris*, *An. sawadwongporni*, *An. dirus*, and *An. epiroticus* [156]. Beyond effects on survival, ivermectin inhibits the sporogony of *P. vivax* in *An. dirus* and *An. minimus* at human-relevant concentrations [156, 157]. Primary metabolites (e.g., M1, M3, M6) also exhibit mosquito-lethal effects, potentially extending mortality beyond that of the parent compound [157]. However, recent research indicates that the B1a and B1b components of ivermectin have no effect on mosquito mortality [158].

Genetic control is a key frontier in eradication, with research focused on molecular targets to block transmission in *An. dirus* [113]. The suppressor of cytokine signalling (SOCS) gene is activated in the abdomen 12–24 h after an infectious *P. vivax* blood meal, indicating involvement in the immune response [159]. The conserved HPX15 gene may facilitate parasite invasion in *Anopheles*, making it a potential target [160]. Targeting the yellow-g gene in *An. dirus* reduces *P. vivax* infection without shortening lifespan, a desirable profile for control strategies [161]. Carboxypeptidase B in the midgut plays a vital role in *Plasmodium* sexual development; compounds such as NSC-1014, NSC-332670, and aminopterin significantly reduced carboxypeptidase B activity in *An. minimus*, leading to reduced parasite infection [162].

Monitoring insecticide resistance is central to sustaining the effectiveness of chemical vector control [163] within a One Health framework for zoonotic malaria. Longitudinal molecular and phenotypic evidence from Thailand indicates that, among primary malaria vectors implicated in *P. knowlesi* transmission, target-site resistance to pyrethroids remains rare. Molecular screening for target-site resistance in eight *Anopheles* species (*An. baimaii*, *An. minimus* s.s., *An. epiroticus*, *An. jamesii*, *An. maculatus* s.s., *An. barbirostris* A3, *An. sawadwongporni*, and *An. aconitus*) from Ranong Province, a *P. knowlesi*-endemic area in Thailand, detected only the wild-type L1014 allele in the voltage-gated sodium channel gene [37]. Consistent with these findings, WHO susceptibility bioassays conducted across multiple regions demonstrated high levels of pyrethroid sensitivity in key vector species, *An. minimus* s.l. and *An. maculatus* s.l. from Tak Province, *An minimus* and *An aconitus* from Kanchanaburi and Ratchaburi provinces [105], *An dirus* from Trat Province [11], and *An epiroticus* from coastal provinces (Trat, Songkhla, Surat Thani, and Phang Nga) [183], with 24-h mortality frequently reaching 98–100% following exposure to deltamethrin (0.05%), permethrin (0.75%), lambda-cyhalothrin (0.05%), cyfluthrin (1.5%), and etofenprox (0.5%) [164].

Despite this overall susceptibility, emerging resistance patterns were observed in selected secondary vectors and for nonpyrethroid insecticide classes. In Tak Province (western Thailand), reduced mortality to 0.1% bendiocarb was detected in *An. minimus* s.l. (88.8% mortality) and *An. maculatus* s.l. (89.9% mortality), while susceptibility to propoxur (0.1%) and organophosphates (fenitrothion 1.0% and malathion 5.0%) remained intact [164]. While field populations of *An. dirus* s.l. and *An. maculatus* s.l. from northeastern Thailand in Ubon Ratchathani Province including *An. harrisoni* and *An. dirus* from Kanchanaburi were fully susceptible to deltamethrin and transfluthrin (discriminating concentrations (0.06% for *An. minimus*; 0.02% for *An. dirus*) [184], secondary vectors (*An peditaeniatus* and *An barbirostris* s.l.) in Ubon Ratchathani exhibited resistance or suspected resistance to DDT and, in some cases, pyrethroids (e.g., deltamethrin 0.05%, permethrin 0.75%) [3, 43], indicative of metabolic rather than target-site mechanisms [43]. Synergist assays using piperonyl butoxide restored pyrethroid susceptibility in *An. peditaeniatus*, implicating cytochrome P450-mediated detoxification, and *kdr* mutations were absent in secondary vector populations examined to date [43]. *An. nivipes* and *An. philippinensis* remained fully susceptible to these insecticides [43].

Behavioral responses further complicate vector control measures. Strong contact irritancy and moderate noncontact repellency to pyrethroids (deltamethrin, permethrin, and alpha-cypermethrin) were documented in *An. epiroticus* from Chang Island (Trat Province), potentially reducing intervention effectiveness despite physiological susceptibility [183]. Similarly, exophagic and outdoor-resting behaviors observed in *An. dirus* and *An. minimus* populations associated with rubber plantation landscapes may limit the impact of indoor-based interventions such as IRS and ITN/LLINs [11].

Laboratory studies also indicate that mosquito age (3–5 days versus 20–23 days) and batch size (5, 10, or 20 individuals) significantly influence mortality and knockdown outcomes in WHO tube assays, particularly for volatile pyrethroids such as transfluthrin [165]. Adaptations to testing protocols, including the use of smaller batch sizes with increased replication, can improve data reliability when wild mosquito numbers are limited [165], while remaining consistent with WHO recommendation of 25 nonfed females that are 3–5 days old [166].

Consistent with a One Health approach, these findings highlight the need to align resistance surveillance and vector control strategies with landscape-driven transmission dynamics. In settings where zoonotic malaria is sustained at the interface of humans, NHP reservoirs, and forest-associated vectors, effective control cannot rely on single interventions or narrowly defined indicators of success. Addressing *P knowlesi* and other simian malaria parasites requires a One Health approach that recognizes how land-use change, macaque ecology, vector behavior, and insecticide susceptibility interact to shape transmission risk. Strengthening integrated surveillance across these domains, and translating this evidence into adaptive, context-specific vector control strategies, will be essential for mitigating spillover risk and sustaining malaria control gains in rapidly changing landscapes.

## Conclusions

Spatial and behavioral heterogeneity among *Anopheles* vectors, including variation in host preference, biting time, and resting behavior, strongly shapes malaria transmission dynamics and the effectiveness of control interventions. These traits are further modified by environmental change and human activities, underscoring the need for adaptive, evidence-based surveillance within national malaria control programs. Although major vectors account for most current transmission in Thailand, the potential contribution of secondary vectors should not be overlooked. Accordingly, the use of complementary collection and monitoring approaches is essential to characterize human and vector behavioral diversity, detect early behavioral shifts. and sustain control effectiveness [167].

A persistent challenge is the incomplete understanding of sibling species diversity, geographic distribution, and sympatry within major vector complexes, particularly the Barbirostris, Minimus, Maculatus, and Hyrcanus groups. Closely related species can differ markedly in vectorial capacity, host preference, biting and resting behavior, and insecticide susceptibility, such that interventions effective against one species may be less effective against another[ 168, 170, 171]. Progress is constrained by limitations of existing molecular identification tools, which may yield ambiguous or incorrect results, while newer assays such as Dirus Complex species identification PCR (DiCSIP) with improved resolution remain underutilized [32]. These gaps are further compounded by limited ecological data on climate variability, larval habitats, forest microclimates, land-use change, and the effects of geographic isolation on gene flow, local adaptation, and resistance emergence.

Within this context, this review synthesizes recent advances in molecular biosystematics, bionomics, and vector incrimination of *Anopheles* complexes in Thailand to clarify their roles in human and zoonotic malaria within a One Health framework. ITS2- and mitochondrial DNA-based diagnostics have resolved cryptic sibling species, updated the distributions of primary vectors (Dirus, Minimus, and Maculatus) and key secondary groups (Barbirostris, Sundaicus, Hyrcanus, and Subpictus), revealing substantial genetic and ecological diversification across forest—agricultural mosaics and international border regions.

Evidence indicates highly heterogeneous infection prevalences, expanding assemblages of NHP malaria *Plasmodium* maintained predominantly by Leucosphyrus-group vectors, and marked behavioral plasticity—including early, outdoor, and zoophilic biting—that sustains residual and zoonotic transmission despite generally high pyrethroid susceptibility. Emerging nonpyrethroid resistance, cryptic larval habitats, and ongoing land-use change further complicate vector control and expose critical gaps in human–vector–macaque contact patterns and vector competence.

Overall, addressing residual and zoonotic malaria transmission in Thailand will require integrated, species-specific surveillance that links molecular identification, vector behavior, host ecology, and environmental change. Such an approach, consistent with a One Health framework, is essential to inform adaptive, landscape-specific vector control strategies as Thailand progress toward malaria elimination and confronts the growing challenge of *P. knowlesi* and other zoonotic malaria threats.

## Data Availability

The data supporting the contents of this article are included within the article.

## Abbreviations

AS-PCR: Allele-specific polymerase chain reaction
CBT: Cattle-baited traps
CDC: Centers for Diseases Control and Prevention
DiCSIP: Dirus complex species identification polymerase chain reaction
DDT: Dichlorodiphenyltrichloroethane
GMS: Greater Mekong Subregion
HLC: Human landing catch
IRS: Indoor residual spraying
ITS2: Internal transcribed spacer 2
NHP: Nonhuman primate
PCR: Polymerase chain reaction
PRISMA: Preferred Reporting Items for Systematic Reviews and Meta-Analyses
RFLP-PCR: Polymerase chain reaction with restriction fragment length polymorphism
SCAR-PCR: Sequence characterized amplified region-polymerase chain reaction
WHO: World Health Organization

## References

[1] Tananchai C, Manguin S, Bangs MJ, Chareonviriyaphap T. Malaria vectors and species complexes in Thailand: implications for vector control. Trends Parasitol. 2019;35:544–58. 10.1016/j.pt.2019.04.013. 31182384 10.1016/j.pt.2019.04.013

[2] Yanmanee S, Seethamchai S, Kuamsab N, Tia T, Jongwutiwes S, Putaporntip C. *Anopheles* population belonging to Barbirostris complex in Narathiwat Province and its vectorial status for human and simian malaria. Chulalongkorn Medical Journal. 2024;67:297-308. 10.56808/2673-060x.5368.

[3] Sumarnrote A, Overgaard HJ, Corbel V, Thanispong K, Chareonviriyaphap T, Manguin S. Species diversity and insecticide resistance within the *Anopheles hyrcanus* group in Ubon Ratchathani Province, Thailand. Parasit Vectors. 2020;13:525. 10.1186/s13071-020-04389-4. 33069255 10.1186/s13071-020-04389-4 PMC7568835

[4] https://www.who.int/news-room/feature-stories/detail/thailand-gears-up-to-eliminatemalaria-by-2024.

[5] World Health Organization. World malaria report 2024: addressing inequity in the global malaria response. Geneva: World Health Organization; 2024.

[6] Thailand Malaria Elimination Project. https://malaria.ddc.moph.go.th/. Accessed 12 Sep 2025.

[7] Araki T, Koyama A, Yoshimura H, Arai A, Kawai S, Sekizawa S, et al. Ultrasensitive malaria detection system for *Anopheles* mosquito field surveillance using droplet digital PCR. Parasitol Int. 2024;101:102891. 10.1016/j.parint.2024.102891. 38537686 10.1016/j.parint.2024.102891

[8] Fornace KM, Abidin TR, Alexander N, Brock P, Grigg MJ, Murphy A, et al. Association between landscape factors and spatial patterns of *Plasmodium knowlesi* infections in Sabah, Malaysia. Emerg Infect Dis. 2016;22:201–8. 10.3201/eid2202.150656. 26812373 10.3201/eid2202.150656 PMC4734530

[9] Moyes CL, Shearer FM, Huang Z, Wiebe A, Gibson HS, Nijman V, et al. Predicting the geographical distributions of the macaque hosts and mosquito vectors of *Plasmodium knowlesi* malaria in forested and non-forested areas. Parasit Vectors. 2016;9:242. 10.1186/s13071-016-1527-0. 27125995 10.1186/s13071-016-1527-0 PMC4850754

[10] van de Straat B, Sebayang B, Grigg MJ, Staunton K, Garjito TA, Vythilingam I, et al. Zoonotic malaria transmission and land use change in Southeast Asia: what is known about the vectors. Malar J. 2022;21:109. 10.1186/s12936-022-04129-2. 35361218 10.1186/s12936-022-04129-2 PMC8974233

[11] Pimnon S, Bhumiratana A. Adaptation of *Anopheles* Vectors to Anthropogenic Malaria-Associated Rubber Plantations and Indoor Residual Spraying: Establishing Population Dynamics and Insecticide Susceptibility. Can J Infect Dis Med Microbiol. 2018;2018 9853409:1-17. 10.1155/2018/9853409. 30034563 10.1155/2018/9853409 PMC6032653

[12] Byrne I, Aure W, Manin BO, Vythilingam I, Ferguson HM, Drakeley CJ, et al. Environmental and spatial risk factors for the larval habitats of *Plasmodium knowlesi* vectors in Sabah, Malaysian Borneo. Sci Rep. 2021;11:11810. 10.1038/s41598-021-90893-1. 34083582 10.1038/s41598-021-90893-1 PMC8175559

[13] Jeyaprakasam NK, Liew JWK, Low VL, Wan-Sulaiman WY, Vythilingam I. *Plasmodium knowlesi* infecting humans in Southeast Asia: what’s next? PLoS Negl Trop Dis. 2020;14:e0008900. 10.1371/journal.pntd.0008900. 33382697 10.1371/journal.pntd.0008900 PMC7774830

[14] Akter R, Vythilingam I, Khaw LT, Qvist R, Lim YA, Sitam FT, et al. Simian malaria in wild macaques: first report from Hulu Selangor district, Selangor, Malaysia. Malar J. 2015;14:386. 10.1186/s12936-015-0856-3. 26437652 10.1186/s12936-015-0856-3 PMC4595055

[15] Hii J, Rueda LM. Malaria vectors in the Greater Mekong Subregion: overview of malaria vectors and remaining challenges. Southeast Asian J Trop Med Public Health. 2013;44:73–165. 24159831

[16] Tainchum K, Kongmee M, Manguin S, Bangs MJ, Chareonviriyaphap T. *Anopheles* species diversity and distribution of the malaria vectors of Thailand. Trends Parasitol. 2015;31:109–19. 10.1016/j.pt.2015.01.004. 25697632 10.1016/j.pt.2015.01.004

[17] Manguin S, Garros C, Dusfour I, Harbach RE, Coosemans M. Bionomics, taxonomy, and distribution of the major malaria vector taxa of *Anopheles* subgenus Cellia in Southeast Asia: an updated review. Infect Genet Evol. 2008;8:489–503. 10.1016/j.meegid.2007.11.004. 18178531 10.1016/j.meegid.2007.11.004

[18] Harrison BA. Medical Entomology Studies-XIII. The Myzomyia Series of *Anopheles* (Cellia) in Thailand, with Emphasis on Intra-Interspecific Variations (Diptera: Culicidae). Contrib Am Entomol Inst. 1980;17:1-195.

[19] Rattanarithikul R, Harrison BA. An illustrated key to the *Anopheles* larvae of Thailand. United States Army Medical Component, South East Asia Treaty Organization; 1973.

[20] Rattanarithikul R, Harrison BA, Harbach RE, Panthusiri P, Coleman RE, Panthusiri P. Illustrated keys to the mosquitoes of Thailand. IV. *Anopheles*. Southeast Asian J Trop Med Public Health. 2006;37:1–128. 17262930

[21] Harrison B, Scanlon J. Medical entomology studies-II. The subgenus *Anopheles* in Thailand (Diptera: Culicidae). 1975.

[22] Baimai V, Green CA, Andre RG, Harrison BA, Peyton EL. Cytogenetic studies of some species complexes of *Anopheles* in Thailand and Southeast Asia. Southeast Asian J Trop Med Public Health. 1984;15:536–46. 6543543

[23] Baimai V. Population cytogenetics of the malaria vector *Anopheles leucosphyrus* group. Southeast Asian J Trop Med Public Health. 1988;19:667–80. 3238480

[24] Baimai V, Poopittayasataporn A, Kijchalao U. Cytological differences and chromosomal rearrangements in four members of the *Anopheles dirus* complex (Diptera: Culicidae). Genome. 1988;30:372–9. 10.1139/g88-065. 3169545 10.1139/g88-065

[25] Baimai V, Treesucon A, Kijchalao U. Heterochromatin variation in chromosome X in a natural population of *Anopheles willmori* (Diptera: Culicidae) of Thailand. Genetica. 1996;97:235–9. 10.1007/bf00054630. 8901140 10.1007/BF00054630

[26] Baimai V, Rattanarithikul R, Kijchalao U. Metaphase karyotypes of *Anopheles* of Thailand and Southeast Asia: I. the Hyrcanus Group. J Am Mosq Control Assoc. 1993;9:59–67. 8468575

[27] Baimai V, Rattanarithikul R, Kijchalao U. Metaphase karyotypes of *Anopheles* of Thailand and Southeast Asia: IV. the Barbirostris and Umbrosus species groups, subgenus *Anopheles* (Diptera: Culicidae). J Am Mosq Control Assoc. 1995;11:323–8. 8551301

[28] Manguin S, Kengne P, Sonnier L, Harbach RE, Baimai V, Trung HD, et al. SCAR markers and multiplex PCR-based identification of isomorphic species in the *Anopheles dirus* complex in Southeast Asia. Med Vet Entomol. 2002;16:46–54. 10.1046/j.0269-283x.2002.00344.x. 11963981 10.1046/j.0269-283x.2002.00344.x

[29] Eamkum P, Sungvornyothin S, Kritpetcharat O, Daduang J, Lek-Uthai U, Charerntanyarak L, et al. A single-round multiplex PCR assay for the identification of *Anopheles minimus* related species infected with *Plasmodium falciparum* and *Plasmodium vivax*. Parasitol Int. 2014;63:442–9. 10.1016/j.parint.2013.11.001. 24239524 10.1016/j.parint.2013.11.001

[30] Brosseau L, Udom C, Sukkanon C, Chareonviriyaphap T, Bangs MJ, Saeung A, et al. A multiplex PCR assay for the identification of five species of the *Anopheles barbirostris* complex in Thailand. Parasit Vectors. 2019;12:223. 10.1186/s13071-019-3494-8. 31088534 10.1186/s13071-019-3494-8 PMC6515612

[31] Wilai P, Namgay R, Made Ali RS, Saingamsook J, Saeung A, Junkum A, et al. A multiplex PCR based on mitochondrial COI sequences for identification of members of the *Anopheles barbirostris* complex (Diptera: Culicidae) in Thailand and other countries in the region. Insects. 2020;11:7. 10.3390/insects11070409. 10.3390/insects11070409 PMC7412068 32630637

[32] Saeung M, Pengon J, Pethrak C, Thaiudomsup S, Lhaosudto S, Saeung A, et al. Dirus complex species identification PCR (DiCSIP) improves the identification of *Anopheles dirus* complex from the Greater Mekong Subregion. Parasit Vectors. 2024;17:260. 10.1186/s13071-024-06321-6. 38880909 10.1186/s13071-024-06321-6 PMC11181648

[33] Page MJ, McKenzie JE, Bossuyt PM, Boutron I, Hoffmann TC, Mulrow CD, et al. The PRISMA 2020 statement: an updated guideline for reporting systematic reviews. BMJ. 2021;372:n71. 10.1136/bmj.n71. 33782057 10.1136/bmj.n71 PMC8005924

[34] https://www.prisma-statement.org/prisma-2020-flow-diagram. Accessed 05 Apr 2025.

[35] Balduzzi S, Rucker G, Schwarzer G. How to perform a meta-analysis with R: a practical tutorial. Evid Based Ment Health. 2019;22:153–60. 10.1136/ebmental-2019-300117. 31563865 10.1136/ebmental-2019-300117 PMC10231495

[36] Sukkanon C, Masangkay FR, Mala W, Kotepui KU, Wilairatana P, Chareonviriyaphap T, et al. Prevalence of *Plasmodium* spp. in *Anopheles* mosquitoes in Thailand: a systematic review and meta-analysis. Parasit Vectors. 2022;15:285. 10.1186/s13071-022-05397-2. 35933389 10.1186/s13071-022-05397-2 PMC9357324

[37] Chaiphongpachara T, Laojun S, Changbunjong T, Sumruayphol S, Suwandittakul N, Chookaew S, et al. Genetic Diversity, Haplotype Relationships, and kdr Mutation of Malaria Anopheles Vectors in the Most Plasmodium knowlesi-Endemic Area of Thailand. Trop Med Infect Dis. 2022;7 412:1-17. 10.3390/tropicalmed7120412. 36548667 10.3390/tropicalmed7120412 PMC9786164

[38] Walton C, Somboon P, O’Loughlin SM, Zhang S, Harbach RE, Linton YM, et al. Genetic diversity and molecular identification of mosquito species in the *Anopheles maculatus* group using the ITS2 region of rDNA. Infect Genet Evol. 2007;7:93–102. 10.1016/j.meegid.2006.05.001. 16782411 10.1016/j.meegid.2006.05.001

[39] Garros C, Marchand RP, Quang NT, Hai NS, Manguin S. First record of *Anopheles minimus* C and significant decrease of *An. minimus* A in central Vietnam. J Am Mosq Control Assoc. 2005;21:139–43. 10.2987/8756-971X(2005)21[139:FROAMC]2.0.CO;2. 16033115 10.2987/8756-971X(2005)21[139:FROAMC]2.0.CO;2

[40] Chaiphongpachara T, Changbunjong T, Laojun S, Nutepsu T, Suwandittakul N, Kuntawong K, et al. Mitochondrial DNA barcoding of mosquito species (Diptera: Culicidae) in Thailand. PLoS ONE. 2022;17 9:e275090. 10.1371/journal.pone.0275090. 36137118 10.1371/journal.pone.0275090 PMC9642330

[41] Chaiphongpachara T, Changbunjong T, Laojun S, Sumruayphol S, Suwandittakul N, Kuntawong K, et al. Geometric morphometric and molecular techniques for discriminating among three cryptic species of the *Anopheles barbirostris* complex (Diptera: Culicidae) in Thailand. Heliyon. 2022;8:e11261. 10.1016/j.heliyon.2022.e11261. 36339998 10.1016/j.heliyon.2022.e11261 PMC9634016

[42] Hempolchom C, Yasanga T, Wijit A, Taai K, Dedkhad W, Srisuka W, et al. Scanning electron microscopy of antennal sensilla of the eight *Anopheles* species of the Hyrcanus Group (Diptera: Culicidae) in Thailand. Parasitol Res. 2017;116:143–53. 10.1007/s00436-016-5270-4. 27752768 10.1007/s00436-016-5270-4

[43] Sumarnrote A, Overgaard HJ, Marasri N, Fustec B, Thanispong K, Chareonviriyaphap T, et al. Status of insecticide resistance in *Anopheles* mosquitoes in Ubon Ratchathani province, Northeastern Thailand. Malar J. 2017;16:299. 10.1186/s12936-017-1948-z. 28743278 10.1186/s12936-017-1948-z PMC5526291

[44] Bunmee K, Thaenkham U, Saralamba N, Ponlawat A, Zhong D, Cui L, et al. Population genetic structure of the malaria vector *Anopheles minimus* in Thailand based on mitochondrial DNA markers. Parasit Vectors. 2021;14:496. 10.1186/s13071-021-04998-7. 34565456 10.1186/s13071-021-04998-7 PMC8474755

[45] Laojun S, Chaiphongpachara T. Island mosquitoes of Thailand: an update on species diversity and DNA barcoding. Parasitol Res. 2024;123:224. 10.1007/s00436-024-08237-7. 38809447 10.1007/s00436-024-08237-7

[46] Poolphol P, Harbach RE, Sriwichai P, Aupalee K, Sattabongkot J, Kumpitak C, et al. Natural *Plasmodium vivax* infections in *Anopheles* mosquitoes in a malaria endemic area of northeastern Thailand. Parasitol Res. 2017;116:3349–59. 10.1007/s00436-017-5653-1. 29082435 10.1007/s00436-017-5653-1

[47] Tananchai C, Pattanakul M, Nararak J, Sinou V, Manguin S, Chareonviriyaphap T. Diversity and biting patterns of *Anopheles* species in a malaria endemic area, Umphang Valley, Tak Province, western Thailand. Acta Trop. 2019;190:183–92. 10.1016/j.actatropica.2018.11.009. 30439344 10.1016/j.actatropica.2018.11.009

[48] Gingrich JB, Weatherhead A, Sattabongkot J, Pilakasiri C, Wirtz RA. Hyperendemic malaria in a Thai village: dependence of year-round transmission on focal and seasonally circumscribed mosquito (Diptera: Culicidae) habitats. J Med Entomol. 1990;27:1016–26. 10.1093/jmedent/27.6.1016. 2280385 10.1093/jmedent/27.6.1016

[49] Bhumiratana A, Sorosjinda-Nunthawarasilp P, Kaewwaen W, Maneekan P, Pimnon S. Malaria-associated rubber plantations in Thailand. Travel Med Infect Dis. 2013;11:37–50. 23200406 10.1016/j.tmaid.2012.11.002

[50] Chujun S, Chaivisit P, Chutinantakul A. Epidemiological characteristics and factors related to malarial disease in Thai and migrant patients in the upper part of Southern Thailand. Dis Control J. 2019. 10.1186/s13071-021-04870-8.

[51] Wamaket N, Khamprapa O, Chainarin S, Thamsawet P, Ninsaeng U, Thongsalee S, et al. *Anopheles* bionomics in a malaria endemic area of southern Thailand. Parasit Vectors. 2021;14:378. 10.1186/s13071-021-04870-8. 34315509 10.1186/s13071-021-04870-8 PMC8317318

[52] Saeung M, Jupatanakul N, Afelt A, Suksirisawat K, Lhaosudto S, Ahebwa A, et al. Insights into spatio-temporal dynamics of *Anopheles* vectors while approaching malaria elimination along the Thailand-Cambodia border. Acta Trop. 2025;263:107545. 10.1016/j.actatropica.2025.107545. 39933646 10.1016/j.actatropica.2025.107545

[53] Rattanarithikul R, Green CA, Panyim S, Noigamol C, Chanaimongkol S, Mahapibul P. Larval habitats of malaria vectors and other *Anopheles* mosquitoes around a transmission focus in Northwestern Thailand. J Am Mosq Control Assoc. 1995;11:428–33. 8825502

[54] Rosenberg R, Andre RG, Ketrangsee S. Seasonal fluctuation of *Plasmodium falciparum* gametocytaemia. Trans R Soc Trop Med Hyg. 1990;84:29–33. 10.1016/0035-9203(90)90369-p. 2189241 10.1016/0035-9203(90)90369-p

[55] Laojun S, Changbunjong T, Chaiphongpachara T. Insights into the mitochondrial cytochrome oxidase I (mt-COI) gene and wing morphometrics of *Anopheles baimaii* (Diptera: Culicidae) in malaria-endemic islands of Thailand. Parasitol Res. 2024;123:171. 10.1007/s00436-024-08195-0. 38530429 10.1007/s00436-024-08195-0

[56] Obsomer V, Defourny P, Coosemans M. The *Anopheles dirus* complex: spatial distribution and environmental drivers. Malar J. 2007;6:26. 10.1186/1475-2875-6-26. 17341297 10.1186/1475-2875-6-26 PMC1838916

[57] Fantini B. Anophelism without malaria: an ecological and epidemiological puzzle. Parassitologia. 1994;36:83–106. 7898963

[58] Tainchum K, Ritthison W, Chuaycharoensuk T, Bangs MJ, Manguin S, Chareonviriyaphap T. Diversity of *Anopheles* species and trophic behavior of putative malaria vectors in two malaria endemic areas of northwestern Thailand. J Vector Ecol. 2014;39:424–36. 10.1111/jvec.12118. 25424272 10.1111/jvec.12118

[59] Tongkrajang N, Ruenchit P, Tananchai C, Chareonviriyaphap T, Kulkeaw K. Molecular identification of native *Wolbachia pipientis* in *Anopheles minimus* in a low-malaria transmission area of Umphang Valley along the Thailand-Myanmar border. Parasit Vectors. 2020;13:579. 10.1186/s13071-020-04459-7. 33198811 10.1186/s13071-020-04459-7 PMC7670599

[60] Sinka ME, Bangs MJ, Manguin S, Chareonviriyaphap T, Patil AP, Temperley WH, et al. The dominant *Anopheles* vectors of human malaria in the Asia-Pacific region: occurrence data, distribution maps and bionomic precis. Parasit Vectors. 2011;4:89. 10.1186/1756-3305-4-89. 21612587 10.1186/1756-3305-4-89 PMC3127851

[61] Firmansyah NE, Thongseesuksai T, Boonmars T, Laummaunwai P. Investigation of malaria vectors *Anopheles* in non-endemic areas of Thailand: in proximity to workplaces housing foreign migrant workers. Malar J. 2025;24:18. 10.1186/s12936-025-05253-5. 39833851 10.1186/s12936-025-05253-5 PMC11744975

[62] Kittayapong P, Baisley KJ, Baimai V, O’Neill SL. Distribution and diversity of *Wolbachia* infections in Southeast Asian mosquitoes (Diptera: Culicidae). J Med Entomol. 2000;37:340–5. 10.1603/0022-2585(2000)037[0340:Dadowi]2.0.Co;2. 15535575 10.1093/jmedent/37.3.340

[63] Tisgratog R, Tananchai C, Bangs MJ, Tainchum K, Juntarajumnong W, Prabaripai A, et al. Chemically induced behavioral responses in *Anopheles minimus* and *Anopheles harrisoni* in Thailand. J Vector Ecol. 2011;36:321–31. 10.1111/j.1948-7134.2011.00172.x. 22129403 10.1111/j.1948-7134.2011.00172.x

[64] Taai K, Harbach RE, Aupalee K, Srisuka W, Yasanga T, Otsuka Y, et al. An effective method for the identification and separation of *Anopheles minimus*, the primary malaria vector in Thailand, and its sister species *Anopheles harrisoni*, with a comparison of their mating behaviors. Parasit Vectors. 2017;10:97. 10.1186/s13071-017-2035-6. 28222787 10.1186/s13071-017-2035-6 PMC5320799

[65] Poolprasert P, Manguin S, Bangs MJ, Sukhontabhirom S, Poolsomboon S, Akaratanakul P, et al. Genetic structure and gene flow of *Anopheles minimus* and *Anopheles harrisoni* in Kanchanaburi Province, Thailand. J Vector Ecol. 2008;33:158–65. 10.3376/1081-1710(2008)33[158:gsagfo]2.0.co;2. 18697319 10.3376/1081-1710(2008)33[158:gsagfo]2.0.co;2

[66] Chatpiyaphat K, Sumruayphol S, Dujardin JP, Samung Y, Phayakkaphon A, Cui L, et al. Geometric morphometrics to distinguish the cryptic species *Anopheles minimus* and *An. harrisoni* in malaria hot spot villages, western Thailand. Med Vet Entomol. 2021;35:293–301. 10.1111/mve.12493. 33205850 10.1111/mve.12493 PMC8451769

[67] Ma Y, Li S, Xu J. Molecular identification and phylogeny of the Maculatus group of *Anopheles* mosquitoes (Diptera: Culicidae) based on nuclear and mitochondrial DNA sequences. Acta Trop. 2006;99:272–80. 10.1016/j.actatropica.2006.09.005. 17052677 10.1016/j.actatropica.2006.09.005

[68] Somboon P, Thongwat D, Harbach RE. *Anopheles* (Cellia) *rampae* n. sp. alias chromosomal form K of the Oriental Maculatus Group (Diptera: Culicidae) in Southeast Asia. Zootaxa. 2011. 10.1646/zootaxa.2810.1.5.

[69] Sumruayphol S, Chaiphongpachara T, Samung Y, Ruangsittichai J, Cui L, Zhong D, et al. Seasonal dynamics and molecular differentiation of three natural *Anopheles* species (Diptera: Culicidae) of the Maculatus Group (Neocellia Series) in malaria hotspot villages of Thailand. Parasit Vectors. 2020;13:574. 10.1186/s13071-020-04452-0. 33176862 10.1186/s13071-020-04452-0 PMC7659066

[70] Inthitanon N, Sripoorote P, Wattanagoon Y, Petchvijit P, Anantjitsupha A, Win KM, et al. Malaria control among Myanmar migrants in Thailand: a qualitative study of healthcare providers. Malar J. 2025;24:160. 10.1186/s12936-025-05397-4. 40405144 10.1186/s12936-025-05397-4 PMC12096533

[71] World Health Organization. World malaria report 2023. Geneva: World Health Organization; 2023.

[72] Kwansomboon N, Chaumeau V, Kittiphanakun P, Cerqueira D, Corbel V, Chareonviriyaphap T. Vector bionomics and malaria transmission along the Thailand-Myanmar border: a baseline entomological survey. J Vector Ecol. 2017;42:84–93. 10.1111/jvec.12242. 28504441 10.1111/jvec.12242

[73] Marasri N, Overgaard HJ, Sumarnrote A, Thanispong K, Corbel V, Chareonviriyaphap T. Abundance and distribution of *Anopheles* mosquitoes in a malaria endemic area along the Thai-Lao border. J Vector Ecol. 2017;42:325–34. 10.1111/jvec.12273. 29125244 10.1111/jvec.12273

[74] Lyttleton C. Deviance and resistance: Malaria elimination in the Greater Mekong Subregion. Soc Sci Med. 2016;150:144–52. 10.1016/j.socscimed.2015.12.033. 26751710 10.1016/j.socscimed.2015.12.033

[75] https://stat.bora.dopa.go.th. Accessed 12 Sep 2025.

[76] Sumarnrote A, Corbel V, Overgaard HJ, Celhay O, Marasri N, Fustec B, et al. *Plasmodium* infections in *Anopheles* mosquitoes in Ubon Ratchathani Province, Northeastern Thailand during a malaria outbreak. J Am Mosq Control Assoc. 2018;34:11–7. 10.2987/17-6715.1. 31442122 10.2987/17-6715.1

[77] Yanmanee S, Seethamchai S, Kuamsab N, Karaphan S, Suwonkerd W, Jongwutiwes S, et al. Natural vectors of *Plasmodium knowlesi* and other primate, avian and ungulate malaria parasites in Narathiwat Province, Southern Thailand. Sci Rep. 2023;13:8875. 10.1038/s41598-023-36017-3. 37264067 10.1038/s41598-023-36017-3 PMC10235068

[78] Sriwichai P, Samung Y, Sumruayphol S, Kiattibutr K, Kumpitak C, Payakkapol A, et al. Natural human *Plasmodium* infections in major *Anopheles* mosquitoes in Western Thailand. Parasit Vectors. 2016;9:17. 10.1186/s13071-016-1295-x. 26762512 10.1186/s13071-016-1295-x PMC4712558

[79] Poolphol P, Harbach RE, Sriwichai P, Srisuka W, Aupalee K, Taai K, et al. Diversity, seasonal abundance and biting activity of in relation to climatic factors in Northeastern Thailand. Southeast Asian J Trop Med Public Health. 2017;48:1175–87.

[80] Limrat D, Rojruthai B, Apiwathnasorn C, Samung Y, Prommongkol S. *Anopheles barbirostris/campestris* as a probable vector of malaria in Aranyaprathet, Sa Kaeo Province. Southeast Asian J Trop Med Public Health. 2001;32:739–44. 12041547

[81] Udom C, Thanispong K, Manguin S, Chareonviriyaphap T, Fungfuang W. Trophic behavior and species diversity of the *Anopheles barbirostris* Complex (Diptera: Culicidae) in Thailand. J Med Entomol. 2021;58:2376–84. 10.1093/jme/tjab067. 33942866 10.1093/jme/tjab067

[82] Nguyen AHL, Nugraheni YR, Nguyen TT, Aung A, Narapakdeesakul D, Kaewlamun W, et al. Molecular characterization of anopheline mosquitoes from the goat malaria-endemic areas of Thailand. Med Vet Entomol. 2023;37:381–95. 10.1111/mve.12638. 36598082 10.1111/mve.12638

[83] Phunngam P, Chareonviriyaphap T, Bangs MJ, Arunyawat U. Phylogenetic relationships among malaria vectors and closely related species in Thailand using multilocus DNA sequences. J Am Mosq Control Assoc. 2017;33:91–102. 10.2987/17-6637.1. 28590228 10.2987/17-6637.1

[84] Linton Y-M, Dusfour I, Howard T, Manh ND, Dinh TH, Sochanta T, et al. *Anopheles* (Cellia) *epiroticus* (Diptera: Culicidae), a new malaria vector species in the Southeast Asian Sundaicus Complex. Bull Entomol Res. 2005;95:329–39. 16048681 10.1079/ber2005364

[85] Chaiphongpachara T, Laojun S. Variation over time in wing size and shape of the coastal malaria vector *Anopheles* (Cellia) epiroticus Linton and Harbach (Diptera: Culicidae) in Samut Songkhram. Thailand J Adv Vet Anim Res. 2019;6:208–14. 10.5455/javar.2019.f334. 31453193 10.5455/javar.2019.f334 PMC6702878

[86] Sermwittayawong N, Singh B, Nishibuchi M, Sawangjaroen N, Vuddhakul V. Human *Plasmodium knowlesi* infection in Ranong province, southwestern border of Thailand. Malar J. 2012;11:36. 10.1186/1475-2875-11-36. 22313518 10.1186/1475-2875-11-36 PMC3293766

[87] Ritthison W, Tainchum K, Manguin S, Bangs MJ, Chareonviriyaphap T. Biting patterns and host preference of *Anopheles epiroticus* in Chang Island, Trat Province, eastern Thailand. J Vector Ecol. 2014;39:361–71. 10.1111/jvec.12112. 25424266 10.1111/jvec.12112

[88] Harbach RE: Mosquito Taxonomic Inventory. Mosquito taxonomic inventory. 2024. https://mosquito-taxonomic-inventory.myspecies.info/. Accessed 02 Oct 2025.

[89] Hempolchom C, Otsuka Y, Baimai V, Thongsahuan S, Saeung A, Taai K, et al. Development of a multiplex PCR assay for the identification of eight species members of the Thai Hyrcanus Group (Diptera: Culicidae). Appl Entomol Zool. 2013;48:469–76. 10.1007/s13355-013-0207-1.

[90] Wijit A, Taai K, Dedkhad W, Hempolchom C, Thongsahuan S, Srisuka W, et al. Comparative Studies on the Stenogamous and Eurygamous Behavior of Eight *Anopheles* Species of the Hyrcanus Group (Diptera: Culicidae) in Thailand. Insects. 2016;7 2:1-21. 10.3390/insects7020011 10.3390/insects7020011 PMC4931423 27023618

[91] Nugraheni YR, Arnuphapprasert A, Nguyen TT, Narapakdeesakul D, Nguyen HLA, Poofery J, et al. Myzorhynchus series of *Anopheles* mosquitoes as potential vectors of *Plasmodium bubalis* in Thailand. Sci Rep. 2022;12:5747. 35388073 10.1038/s41598-022-09686-9 PMC8987089

[92] Songsawatkiat S, Baimai V, Thongsahuan S, Otsuka Y, Taai K, Hempolchom C, et al. Evidence to support a conspecific nature of allopatric cytological races of *Anopheles nitidus* (Diptera: Culicidae) in Thailand. J Insect Sci. 2014;14 1:1-9. 10.1093/jisesa/ieu149. 25527592 10.1093/jisesa/ieu149 PMC5657971

[93] Saeung A, Baimai V, Thongsahuan S, Otsuka Y, Srisuka W, Taai K, et al. Cytogenetic, cross-mating and molecular evidence of four cytological races of *Anopheles crawfordi* (Diptera: Culicidae) in Thailand and Cambodia. C R Biol. 2014;337:625–34. 10.1016/j.crvi.2014.08.001. 25444706 10.1016/j.crvi.2014.08.001

[94] Wiwatanaratanabutr I. Geographic distribution of wolbachial infections in mosquitoes from Thailand. J Invertebr Pathol. 2013;114:337–40. 10.1016/j.jip.2013.04.011. 23660513 10.1016/j.jip.2013.04.011

[95] Chaiphongpachara T, Yusuk P, Laojun S, Kunphichayadecha C. Environmental factors associated with mosquito vector larvae in a malaria-endemic area in Ratchaburi Province, Thailand. ScientificWorldJournal. 2018;2018:4519094. 10.1155/2018/4519094. 30662376 10.1155/2018/4519094 PMC6312606

[96] Singh B, Kim Sung L, Matusop A, Radhakrishnan A, Shamsul SS, Cox-Singh J, et al. A large focus of naturally acquired *Plasmodium knowlesi* infections in human beings. Lancet. 2004;363:1017–24. 10.1016/S0140-6736(04)15836-4. 15051281 10.1016/S0140-6736(04)15836-4

[97] Cox-Singh J, Singh B. Knowlesi malaria: newly emergent and of public health importance? Trends Parasitol. 2008;24:406–10. 10.1016/j.pt.2008.06.001. 18678527 10.1016/j.pt.2008.06.001 PMC2843823

[98] Cox-Singh J, Davis TM, Lee K-S, Shamsul SS, Matusop A, Ratnam S, et al. *Plasmodium knowlesi* malaria in humans is widely distributed and potentially life threatening. Clin Infect Dis. 2008;46:165–71. 18171245 10.1086/524888 PMC2533694

[99] Lee KS, Cox-Singh J, Brooke G, Matusop A, Singh B. *Plasmodium knowlesi* from archival blood films: further evidence that human infections are widely distributed and not newly emergent in Malaysian Borneo. Int J Parasitol. 2009;39:1125–8. 10.1016/j.ijpara.2009.03.003. 19358848 10.1016/j.ijpara.2009.03.003 PMC2722692

[100] Imwong M, Madmanee W, Suwannasin K, Kunasol C, Peto TJ, Tripura R, et al. Asymptomatic natural human infections with the simian malaria parasites *Plasmodium cynomolgi* and *Plasmodium knowlesi*. J Infect Dis. 2019;219:695–702. 10.1093/infdis/jiy519. 30295822 10.1093/infdis/jiy519 PMC6376906

[101] Putaporntip C, Kuamsab N, Pattanawong U, Yanmanee S, Seethamchai S, Jongwutiwes S. *Plasmodium cynomolgi* co-infections among symptomatic malaria patients, Thailand. Emerg Infect Dis. 2021;27:590–3. 10.3201/eid2702.191660. 33496236 10.3201/eid2702.191660 PMC7853550

[102] Putaporntip C, Kuamsab N, Seethamchai S, Pattanawong U, Rojrung R, Yanmanee S, et al. Cryptic *Plasmodium inui* and *Plasmodium fieldi* infections among symptomatic malaria patients in Thailand. Clin Infect Dis. 2022;75:805–12. 34971372 10.1093/cid/ciab1060

[103] Kumpitak C, Duangmanee A, Thongyod W, Rachaphaew N, Suansomjit C, Manopwisedjaroen K, et al. Human-to-*Anopheles dirus* mosquito transmission of the anthropozoonotic malaria parasite, *Plasmodium knowlesi*. Parasit Vectors. 2024;17 1:415. 10.1186/s13071-024-06500-5. 39367453 10.1186/s13071-024-06500-5 PMC11451161

[104] Chaiphongpachara T, Changbunjong T, Sumruayphol S, Laojun S, Suwandittakul N, Kuntawong K. Geometric morphometrics versus DNA barcoding for the identification of malaria vectors *Anopheles dirus* and *An. baimaii* in the Thai-Cambodia border. Scientific Reports. 2022;12 1:13236. 10.1038/s41598-022-17646-6. 35918453 10.1038/s41598-022-17646-6 PMC9345986

[105] Fansiri T, Jaichapor B, Pongsiri A, Singkhaimuk P, Khongtak P, Chittham W, et al. Species abundance and density of malaria vectors in Western Thailand and implications for disease transmission. Curr Res Parasitol Vector Borne Dis. 2024;5:100170. 10.1016/j.crpvbd.2024.100170. 38406770 10.1016/j.crpvbd.2024.100170 PMC10885546

[106] Fornace KM, Zorello Laporta G, Vythilingham I, Chua TH, Ahmed K, Jeyaprakasam NK, et al. Simian malaria: a narrative review on emergence, epidemiology and threat to global malaria elimination. Lancet Infect Dis. 2023;23:e520–32. 10.1016/S1473-3099(23)00298-0. 37454671 10.1016/S1473-3099(23)00298-0

[107] Masse RS, Vythilingam I, Fornace K, Othman H, Liu X, Jaafar AJ, et al. Impact of environmental factors on the bionomics of *Anopheles* mosquito vectors of zoonotic malaria: a narrative review. One Health. 2025;21:101141. 10.1016/j.onehlt.2025.101141. 40727445 10.1016/j.onehlt.2025.101141 PMC12301798

[108] Manguin S, Bangs MJ, Pothikasikorn J, Chareonviriyaphap T. Review on global co-transmission of human *Plasmodium* species and *Wuchereria bancrofti* by *Anopheles* mosquitoes. Infect Genet Evol. 2010;10:159–77. 10.1016/j.meegid.2009.11.014. 19941975 10.1016/j.meegid.2009.11.014

[109] Durnez L, Coosemans M. Residual transmission of malaria: an old issue for new approaches. *Anopheles* mosquitoes—new insights into malaria vectors; 2013.

[110] Phasomkusolsil S, Tawong J, Monkanna N, Pantuwatana K, Damdangdee N, Khongtak W, et al. Maintenance of mosquito vectors: effects of blood source on feeding, survival, fecundity, and egg hatching rates. J Vector Ecol. 2013;38:38–45. 10.1111/j.1948-7134.2013.12006.x. 23701605 10.1111/j.1948-7134.2013.12006.x

[111] Marchand RP, Culleton R, Maeno Y, Quang NT, Nakazawa S. Co-infections of *Plasmodium**knowlesi*, *P*. *falciparum*, and *P*. *vivax* among humans and *Anopheles**dirus* Mosquitoes Southern Vietnam. Emerg Infect Dis. 2011. 10.3201/eid1707.101551. 21762577 10.3201/eid1707.101551 PMC3381379

[112] Maeno Y. Molecular epidemiology of mosquitoes for the transmission of forest malaria in south-central Vietnam. Trop Med Health. 2017;45:27. 29046610 10.1186/s41182-017-0065-6 PMC5637239

[113] Mongkol W, Nguitragool W, Sattabongkot J, Kubera A. Blood-induced differential gene expression in *Anopheles dirus* evaluated using RNA sequencing. Med Vet Entomol. 2018;32:399–406. 10.1111/mve.12310. 29885058 10.1111/mve.12310

[114] Cohuet A, Harris C, Robert V, Fontenille D. Evolutionary forces on *Anopheles*: what makes a malaria vector? Trends Parasitol. 2010;26:130–6. 10.1016/j.pt.2009.12.001. 20056485 10.1016/j.pt.2009.12.001

[115] Panthawong A, Sukkanon C, Ngoen-Klan R, Hii J, Chareonviriyaphap T. Forced egg laying method to establish F1 progeny from field populations and laboratory strains of *Anopheles* mosquitoes (Diptera: Culicidae) in Thailand. J Med Entomol. 2021;58:2107–13. 10.1093/jme/tjab105. 34104962 10.1093/jme/tjab105

[116] Bodharamik T, Sungvornyothin S, Juntarajumnong W, Bangs MJ, Arunyawat U. Genetic variation of circadian clock genes in a cavernicolous *Anopheles dirus* (Diptera: Culicidae) in Western Thailand. Agric Nat Res. 2021;55:968–75.

[117] World Health Organization. Larval source management: a supplementary measure for malaria vector control. An operational manual. Geneva: World Health Organization; 2012.

[118] Rhosenberg R, Andre RG, Somchit L. Highly efficient dry season transmission of malaria in Thailand. Trans R Soc Trop Med Hyg. 1990;84:22–8. 2189240 10.1016/0035-9203(90)90367-n

[119] Dutta P, Khan SA, Bhattarcharyya DR, Khan AM, Sharma CK, Mahanta J. Studies on the breeding habitats of the vector mosquito *Anopheles baimai* and its relationship to malaria incidence in Northeastern region of India breeding habitats of and its role in incidence of malaria in Northeastern region of India. EcoHealth. 2010;7:498–506. 10.1007/s10393-010-0337-7. 20652822 10.1007/s10393-010-0337-7

[120] Srisuka W, Sulin C, Sommitr W, Rattanarithikul R, Aupalee K, Saeung A, et al. Mosquito (Diptera: Culicidae) diversity and community structure in Inthanon National Park, Northern Thailand. Insects. 2022;13:814. 10.3390/insects13090814. 36135515 10.3390/insects13090814 PMC9505505

[121] Garros C, Van Bortel W, Trung HD, Coosemans M, Manguin S. Review of the Minimus Complex of *Anopheles*, main malaria vector in Southeast Asia: from taxonomic issues to vector control strategies. Trop Med Int Health. 2006;11:102–14. 10.1111/j.1365-3156.2005.01536.x. 16398761 10.1111/j.1365-3156.2005.01536.x

[122] Chaiphongpachara T, Sumruayphol S. Species diversity and distribution of mosquito vectors in coastal habitats of Samut Songkhram province, Thailand. Trop Biomed. 2017;34:524–32. 33592920

[123] Edwards HM, Sriwichai P, Kirabittir K, Prachumsri J, Chavez IF, Hii J. Transmission risk beyond the village: entomological and human factors contributing to residual malaria transmission in an area approaching malaria elimination on the Thailand-Myanmar border. Malar J. 2019;18:221. 10.1186/s12936-019-2852-5. 31262309 10.1186/s12936-019-2852-5 PMC6604376

[124] Nguyen AHL, Pattaradilokrat S, Kaewlamun W, Kaneko O, Asada M, Kaewthamasorn M. *Myzomyia* and *Pyretophorus* series of *Anopheles* mosquitoes acting as probable vectors of the goat malaria parasite *Plasmodium caprae* in Thailand. Sci Rep. 2023;13:145. 10.1038/s41598-022-26833-4. 36599869 10.1038/s41598-022-26833-4 PMC9812981

[125] van de Straat B, Russell TL, Staunton KM, Sinka ME, Burkot TR. A global assessment of surveillance methods for dominant malaria vectors. Sci Rep. 2021;11:15337. 10.1038/s41598-021-94656-w. 34321525 10.1038/s41598-021-94656-w PMC8319300

[126] World Health Organization. Malaria surveillance, monitoring & evaluation: a reference manual. Geneva: World Health Organization; 2018.

[127] Brown R, Salgado-Lynn M, Jumail A, Jalius C, Chua TH, Vythilingam I, et al. Exposure of primate reservoir hosts to mosquito vectors in Malaysian Borneo. EcoHealth. 2022;19:233–45. 10.1007/s10393-022-01586-8. 35553290 10.1007/s10393-022-01586-8 PMC9276546

[128] Wong ML, Chua TH, Leong CS, Khaw LT, Fornace K, Wan-Sulaiman WY, et al. Seasonal and spatial dynamics of the primary vector of *Plasmodium knowlesi* within a major transmission focus in Sabah, Malaysia. PLoS Negl Trop Dis. 2015;9:e0004135. 10.1371/journal.pntd.0004135. 26448052 10.1371/journal.pntd.0004135 PMC4598189

[129] Jiram AI, Vythilingam I, NoorAzian YM, Yusof YM, Azahari AH, Fong MY. Entomologic investigation of *Plasmodium knowlesi* vectors in Kuala Lipis, Pahang, Malaysia. Malar J. 2012;11:213. 10.1186/1475-2875-11-213. 22727041 10.1186/1475-2875-11-213 PMC3476358

[130] Vythilingam I, Noorazian YM, Huat TC, Jiram AI, Yusri YM, Azahari AH, et al. *Plasmodium knowlesi* in humans, macaques and mosquitoes in Peninsular Malaysia. Parasit Vectors. 2008;1:26. 10.1186/1756-3305-1-26. 18710577 10.1186/1756-3305-1-26 PMC2531168

[131] Chua TH, Manin BO, Daim S, Vythilingam I, Drakeley C. Phylogenetic analysis of simian *Plasmodium* spp. infecting *Anopheles balabacensis* Baisas in Sabah, Malaysia. PLoS Negl Trop Dis. 2017;11:e0005991. 10.1371/journal.pntd.0005991. 28968395 10.1371/journal.pntd.0005991 PMC5638607

[132] Ang JXD, Kadir KA, Mohamad DSA, Matusop A, Divis PCS, Yaman K, et al. New vectors in northern Sarawak, Malaysian Borneo, for the zoonotic malaria parasite, *Plasmodium knowlesi*. Parasit Vectors. 2020;13:472. 10.1186/s13071-020-04345-2. 32933567 10.1186/s13071-020-04345-2 PMC7490903

[133] Ang JXD, Yaman K, Kadir KA, Matusop A, Singh B. New vectors that are early feeders for *Plasmodium knowlesi* and other simian malaria parasites in Sarawak, Malaysian Borneo. Sci Rep. 2021;11:7739. 10.1038/s41598-021-86107-3. 33833272 10.1038/s41598-021-86107-3 PMC8032675

[134] Ismail IA, Phinichpongse S, Boonrasri P. Responses of *Anopheles minimus* to DDT residual spraying in a cleared forested foothill area in central Thailand. Acta Trop. 1978;35:69–82. 25000

[135] Nustsathapana S, Sawasdiwongphorn P, Chitprarop U, Cullen J. The behavior of *Anopheles minimus* Theobald (Diptera: Culicidae) subjected to differing levels of DDT selection pressure in northern Thailand. Bull Entomol Res. 1986;76:303–12.

[136] Nakazawa S, Marchand RP, Quang NT, Culleton R, Manh ND, Maeno Y. *Anopheles dirus* co-infection with human and monkey malaria parasites in Vietnam. Int J Parasitol. 2009;39:1533–7. 10.1016/j.ijpara.2009.08.005. 19703460 10.1016/j.ijpara.2009.08.005

[137] World Health Organization. World malaria report 2022. Geneva: World Health Organization; 2022.

[138] Adugna T, Getu E, Yewhelew D. Parous rate and longevity of anophelines mosquitoes in Bure district, northwestern Ethiopia. PLoS ONE. 2022;17:e0263295. 10.1371/journal.pone.0263295. 35120146 10.1371/journal.pone.0263295 PMC8815865

[139] Yan C, Hii J, Ngoen-Klan R, Saeung M, Chareonviriyaphap T. Semi-field evaluation of human landing catches versus human double net trap for estimating human biting rate of *Anopheles minimus* and *Anopheles harrisoni* in Thailand. PeerJ. 2022;10:e13865. 10.7717/peerj.13865. 36101880 10.7717/peerj.13865 PMC9464434

[140] Vajda ÉA, Saeung M, Ross A, McIver DJ, Tatarsky A, Moore SJ, et al. A semi-field evaluation in Thailand of the use of human landing catches (HLC) versus human-baited double net trap (HDN) for assessing the impact of a volatile pyrethroid spatial repellent and pyrethroid-treated clothing on *Anopheles minimus* landing. Malar J. 2023;22:202. 10.1186/s12936-023-04619-x. 37400831 10.1186/s12936-023-04619-x PMC10318828

[141] Ngoenklan R, Thanh Duong T, Duc Chinh V, Quang Thieu N, Hii J, Bangs MJ, et al. Comparison of vector trapping methods for outdoor biting malaria vector surveillance in Thailand and Vietnam. J Med Entomol. 2022;59:2139–49. 10.1093/jme/tjac147. 36208216 10.1093/jme/tjac147

[142] Ponlawat A, Khongtak P, Jaichapor B, Pongsiri A, Evans BP. Field evaluation of two commercial mosquito traps baited with different attractants and colored lights for malaria vector surveillance in Thailand. Parasit Vectors. 2017;10:378. 10.1186/s13071-017-2315-1. 28784149 10.1186/s13071-017-2315-1 PMC5547504

[143] Jhaiaun P, Panthawong A, Saeung M, Sumarnrote A, Kongmee M, Ngoen-Klan R, Chareonviriyaphap T. Comparing Light-Emitting-Diodes Light Traps for Catching *Anopheles* Mosquitoes in a Forest Setting, Western Thailand. Insects. 2021;12:1076. 10.3390/insects12121076 10.3390/insects12121076 PMC8704415 34940164

[144] Lhaosudto S, Ngoen-Klan R, Meunworn V, Kongmee M, Hii J, Chareonviriyaphap T. Comparison of different spectral ranges of UV-LED lighting for outdoor mosquito trapping in forested area in Thailand. J Med Entomol. 2024;61:1510–8. 10.1093/jme/tjae112. 39213441 10.1093/jme/tjae112

[145] Chaiphongpachara T, Laojun S, Kunphichayadecha C. Effectiveness of mosquito magnets for reducing mosquito (Diptera) populations in coastal areas of Samut Songkhram province. Thailand J Adv Vet Anim Res. 2018;5:426–31. 10.5455/javar.2018.e294. 31453153 10.5455/javar.2018.e294 PMC6702912

[146] Jeyaprakasam NK, Pramasivan S, Liew JWK, Van Low L, Wan-Sulaiman WY, Ngui R, et al. Evaluation of Mosquito Magnet and other collection tools for *Anopheles* mosquito vectors of simian malaria. Parasit Vectors. 2021;14:184. 10.1186/s13071-021-04689-3. 33794965 10.1186/s13071-021-04689-3 PMC8015311

[147] Chaiphongpachara T, Sriwichai P, Samung Y, Ruangsittichai J, Morales Vargas RE, Cui L, et al. Geometric morphometrics approach towards discrimination of three member species of Maculatus group in Thailand. Acta Trop. 2019;192:66–74. 10.1016/j.actatropica.2019.01.024. 30710534 10.1016/j.actatropica.2019.01.024 PMC7110943

[148] Kittichai V, Kaewthamasorn M, Chaiphongpachara T, Laojun S, Saiwichai T, Naing KM, et al. Enhance fashion classification of mosquito vector species via self-supervised vision transformer. Sci Rep. 2024;14:31517. 10.1038/s41598-024-83358-8. 39733133 10.1038/s41598-024-83358-8 PMC11682170

[149] Poinsignon A, Cornelie S, Mestres-Simon M, Lanfrancotti A, Rossignol M, Boulanger D, et al. Novel peptide marker corresponding to salivary protein gSG6 potentially identifies exposure to *Anopheles* bites. PLoS ONE. 2008;3:e2472. 10.1371/journal.pone.0002472. 18575604 10.1371/journal.pone.0002472 PMC2427200

[150] Waitayakul A, Somsri S, Sattabongkot J, Looareesuwan S, Cui L, Udomsangpetch R. Natural human humoral response to salivary gland proteins of *Anopheles* mosquitoes in Thailand. Acta Trop. 2006;98:66–73. 10.1016/j.actatropica.2006.02.004. 16530153 10.1016/j.actatropica.2006.02.004

[151] Brosseau L, Drame PM, Besnard P, Toto JC, Foumane V, Le Mire J, et al. Human antibody response to *Anopheles* saliva for comparing the efficacy of three malaria vector control methods in Balombo, Angola. PLoS ONE. 2012;7:e44189. 10.1371/journal.pone.0044189. 23028499 10.1371/journal.pone.0044189 PMC3454387

[152] Ya-Umphan P, Cerqueira D, Cottrell G, Parker DM, Fowkes FJI, Nosten F, et al. *Anopheles* salivary biomarker as a proxy for estimating *Plasmodium falciparum* malaria exposure on the Thailand-Myanmar Border. Am J Trop Med Hyg. 2018;99:350–6. 10.4269/ajtmh.18-0081. 29869601 10.4269/ajtmh.18-0081 PMC6090370

[153] Ya-Umphan P, Cerqueira D, Parker DM, Cottrell G, Poinsignon A, Remoue F, et al. Use of an *Anopheles* salivary biomarker to assess malaria transmission risk along the Thailand-Myanmar Border. J Infect Dis. 2017;215:396–404. 10.1093/infdis/jiw543. 27932615 10.1093/infdis/jiw543 PMC5853934

[154] Fairbanks EL, Saeung M, Pongsiri A, Vajda E, Wang Y, McIver DJ, et al. Inference for entomological semi-field experiments: Fitting a mathematical model assessing personal and community protection of vector-control interventions. Comput Biol Med. 2024;168:107716. 10.1016/j.compbiomed.2023.107716. 38039890 10.1016/j.compbiomed.2023.107716

[155] Sukkanon C, Nararak J, Bangs MJ, Hii J, Chareonviriyaphap T. Behavioral responses to transfluthrin by *Aedes aegypti*, *Anopheles minimus*, *Anopheles harrisoni*, and *Anopheles dirus* (Diptera: Culicidae). PLoS ONE. 2020;15:e0237353. 10.1371/journal.pone.0237353. 32785255 10.1371/journal.pone.0237353 PMC7423148

[156] Kobylinski KC, Ubalee R, Ponlawat A, Nitatsukprasert C, Phasomkulsolsil S, Wattanakul T, et al. Ivermectin susceptibility and sporontocidal effect in Greater Mekong Subregion *Anopheles*. Malar J. 2017;16:280. 10.1186/s12936-017-1923-8. 28687086 10.1186/s12936-017-1923-8 PMC5501099

[157] Kobylinski KC, Tipthara P, Wamaket N, Chainarin S, Kullasakboonsri R, Sriwichai P, et al. Ivermectin metabolites reduce *Anopheles* survival. Sci Rep. 2023;13:8131. 10.1038/s41598-023-34719-2. 37208382 10.1038/s41598-023-34719-2 PMC10199058

[158] Khemrattrakool P, Hongsuwong T, Tipthara P, Kullasakboonsri R, Phanphoowong T, Sriwichai P, et al. Impact of Ivermectin components on *Anopheles dirus* and *Anopheles minimus* mosquito survival. Parasit Vectors. 2024;17:224. 10.1186/s13071-024-06294-6. 38750608 10.1186/s13071-024-06294-6 PMC11097567

[159] Kittiwattanawong K, Ponlawat A, Boonrotpong S, Nanakorn N, Kongchouy N, Moonmake S, et al. The effect of *Plasmodium vivax* infection on SOCS gene expression in *Anopheles dirus* (Diptera: Culicidae). Trop Biomed. 2020;37:397–408. 33612809

[160] Kajla M, Kakani P, Choudhury TP, Kumar V, Gupta K, Dhawan R, et al. *Anopheles stephensi* Heme Peroxidase HPX15 suppresses midgut immunity to support *Plasmodium* development. Front Immunol. 2017;8:249. 10.3389/fimmu.2017.00249. 28352267 10.3389/fimmu.2017.00249 PMC5348522

[161] Mongkol W, Pomun T, Nguitragool W, Kumpitak C, Duangmanee A, Sattabongkot J, et al. *Anopheles dirus* yellow-g mediates *Plasmodium vivax* infection. Trop Med Int Health. 2021;26:1029–35. 10.1111/tmi.13635. 34089555 10.1111/tmi.13635

[162] Mongkol W, Arunyawat U, Surat W, Kubera A. Active compounds against *Anopheles minimus* carboxypeptidase B for malaria transmission-blocking strategy. J Med Entomol. 2015;52:1322–32. 10.1093/jme/tjv133. 26352934 10.1093/jme/tjv133

[163] Chareonviriyaphap T, Bangs MJ, Suwonkerd W, Kongmee M, Corbel V, Ngoen-Klan R. Review of insecticide resistance and behavioral avoidance of vectors of human diseases in Thailand. Parasites Vectors. 2013;6:280. 10.1186/1756-3305-6-280. 24294938 10.1186/1756-3305-6-280 PMC3850650

[164] Pusawang K, Sattabongkot J, Saingamsook J, Zhong D, Yan G, Somboon P, et al. Insecticide Susceptibility Status of *Anopheles* and *Aedes* Mosquitoes in Malaria and Dengue Endemic Areas, Thai-Myanmar Border. Insects. 2022;13:1035. 10.3390/insects13111035 10.3390/insects13111035 PMC9694411 36354859

[165] Saeung M, Ngoen-Klan R, Yan C, Kerdsawang J, Nararak J, Manguin S, et al. Effects of mosquito age and batch size on knockdown and mortality of laboratory-reared *Anopheles dirus*, *Anopheles minimus*, and wild-caught *Anopheles harrisoni* (Diptera: Culicidae) exposed to transfluthrin using WHO tube and CDC bottle bioassays. J Med Entomol. 2024;61:427–41. 10.1093/jme/tjae004. 38284470 10.1093/jme/tjae004

[166] World Health Organization. Standard operating procedure for testing insecticide susceptibility of adult mosquitoes in WHO tube tests. Geneva: World Health Organization; 2022.

[167] Russell TL, Beebe NW, Cooper RD, Lobo NF, Burkot TR. Successful malaria elimination strategies require interventions that target changing vector behaviors. Malar J. 2013;12:56. 10.1186/1475-2875-12-56. 23388506 10.1186/1475-2875-12-56 PMC3570334

[168] Sinka ME, Golding N, Massey NC, Wiebe A, Huang Z, Hay SI, et al. Modelling the relative abundance of the primary African vectors of malaria before and after the implementation of indoor, insecticide-based vector control. Malar J. 2016;15:142. 10.1186/s12936-016-1187-8. 26945997 10.1186/s12936-016-1187-8 PMC4779559

[169] Boonroumkaew P, Rodpai R, Saeung A, Aupalee K, Saingamsook J, Poolphol P, et al. Bacterial community structure of *Anopheles hyrcanus* group, *Anopheles nivipes*, *Anopheles philippinensis*, and *Anopheles vagus* from a malaria-endemic area in Thailand. PLoS ONE. 2023;18:e0289733. 10.1371/journal.pone.0289733. 37590198 10.1371/journal.pone.0289733 PMC10434920

[170] Edwards HM, Chinh VD, Le Duy B, Thanh PV, Thang ND, Trang DM, et al. Characterising residual malaria transmission in forested areas with low coverage of core vector control in Central Viet Nam. Parasit Vectors. 2019;12:454. 10.1186/s13071-019-3695-1. 31533794 10.1186/s13071-019-3695-1 PMC6751671

[171] Chaiphongpachara T, Laojun S, Changbunjong T, Wichit S, Villarroel PMS. Demographic inference from the mt-DNA COI gene and wing geometry of *Culex gelidus* (Diptera: Culicidae), an important vector of Japanese encephalitis in Thailand. Acta Trop. 2024;256:107276. 10.1016/j.actatropica.2024.107276. 38821146 10.1016/j.actatropica.2024.107276

[172] Sriwichai P, Karl S, Samung Y, Kiattibutr K, Sirichaisinthop J, Mueller I, et al. Imported *Plasmodium falciparum* and locally transmitted *Plasmodium vivax*: cross-border malaria transmission scenario in Northwestern Thailand. Malar J. 2017;16:258. 10.1186/s12936-017-1900-2. 28637467 10.1186/s12936-017-1900-2 PMC5480133

[173] Rattaprasert P, Chaksangchaichot P, Wihokhoen B, Suparach N, Sorosjinda-Nunthawarasilp P. Detection of putative antimalarial-resistant *Plasmodium vivax* in *Anopheles* vectors at Thailand-Cambodia and Thailand-Myanmar borders. Southeast Asian J Trop Med Public Health. 2016;47:182–93. 27244954

[174] Tainchum K, Dupont C, Chareonviriyaphap T, Jumas-Bilak E, Bangs MJ, Manguin S. Bacterial microbiome in wild-caught *Anopheles* mosquitoes in Western Thailand. Front Microbiol. 2020;11:965. 10.3389/fmicb.2020.00965. 32508784 10.3389/fmicb.2020.00965 PMC7253650

[175] Chaiphongpachara T, Laojun S. Wing morphometric variability of the malaria vector *Anopheles* (Cellia) *epiroticus* Linton et Harbach (Diptera: Culicidae) for the duration of the rainy season in coastal areas of Samut Songkhram, Thailand. Folia Parasitologica. 2020;67:007. 10.14411/fp.2020.007. 32350157 10.14411/fp.2020.007

[176] Chaiphongpachara T, Laojuna S. Seasonal species composition, abundance and public health importance of mosquito vectors (Diptera: Culicidae) in Huai Tha Khoei Reservoir, Ratchaburi, Thailand. Biodiversitas J Biol Divers. 2024. 10.13057/biodiv/d250343.

[177] Sanisuriwong J, Yurayart N, Thontiravong A, Tiawsirisup S. Duck Tembusu virus detection and characterization from mosquitoes in duck farms, Thailand. Transbound Emerg Dis. 2020;67:1082–8. 10.1111/tbed.13474. 31913570 10.1111/tbed.13474

[178] Sriwichai P, Karl S, Samung Y, Sumruayphol S, Kiattibutr K, Payakkapol A, et al. Evaluation of CDC light traps for mosquito surveillance in a malaria endemic area on the Thai-Myanmar border. Parasites Vectors. 2015;8:636. 10.1186/s13071-015-1225-3. 26666683 10.1186/s13071-015-1225-3 PMC4678759

[179] Saeung M, Jhaiaun P, Bangs MJ, Ngoen-Klan R, Chareonviriyaphap T. Transmitted light as attractant with mechanical traps for collecting nocturnal mosquitoes in urban Bangkok, Thailand. J Am Mosq Control Assoc. 2021;37:132–42. 10.2987/20-6984.1. 34407172 10.2987/20-6984.1

[180] Thongsripong P, Green A, Kittayapong P, Kapan D, Wilcox B, Bennett S. Mosquito Vector Diversity across Habitats in Central Thailand Endemic for Dengue and Other Arthropod-Borne Diseases. PLoS Neglected Tropical Diseases. 2013;7 10:e2507. 10.1371/journal.pntd.0002507. 24205420 10.1371/journal.pntd.0002507 PMC3814347

[181] Pusawang K, Sriwichai P, Aupalee K, Yasanga T, Phuackchantuck R, Zhong D, et al. Antennal morphology and sensilla ultrastructure of the malaria vectors, *Anopheles maculatus* and *An. sawadwongporni* (Diptera: Culicidae). Arthropod Struct Dev. 2023;76:101296. 10.1016/j.asd.2023.101296. 37657362 10.1016/j.asd.2023.101296 PMC10530502

[182] Chaiphongpachara T. Short communication: a checklist of the mosquito species (Diptera: Culicidae) in the Suan Phueng District, Ratchaburi Province, Thailand. Biodiversitas. 2019;20:468–73. 10.13057/biodiv/d200224.

[183] Ritthison W, Titgratog R, Tainchum K, Bangs MJ, Manguin S, Chareonviriyaphap T. Pyrethroid susceptibility and behavioral avoidance in *Anopheles epiroticus*, a malaria vector in Thailand. J Vector Ecol. 2014;39:32–43. 10.1111/j.1948-7134.2014.12067.x. 24820553 10.1111/j.1948-7134.2014.12067.x

[184] Sukkanon C, Bangs MJ, Nararak J, Hii J, Chareonviriyaphap T. Discriminating lethal concentrations for Transfluthrin, a volatile pyrethroid compound for mosquito control in Thailand. J Am Mosq Control Assoc. 2019;35:258–66. 10.2987/19-6832.1. 31922934 10.2987/19-6832.1

